# Linking Virulence and Iron Limitation Response in : The sRNA IsrR Is Involved in SaeRS Activation

**DOI:** 10.1021/acs.jproteome.5c00059

**Published:** 2025-06-02

**Authors:** Larissa M. Busch, Alexander Ganske, Sebastian Reißel, Lisa Bleul, Christian Hentschker, Hannes Wolfgramm, Leif Steil, Manuela Gesell Salazar, Marc Schaffer, Alexander Reder, Stephan Michalik, Christiane Wolz, Kristin Surmann, Uwe Völker, Ulrike Mäder

**Affiliations:** † Interfaculty Institute for Genetics and Functional Genomics, Department of Functional Genomics, 60634 University Medicine Greifswald, Felix-Hausdorff-Str. 8, 17475 Greifswald, Germany; ‡ Interfaculty Institute of Microbiology and Infection Medicine, 9188 University of Tübingen, Auf der Morgenstelle 28E, 72076 Tübingen, Germany

**Keywords:** *Staphylococcus aureus*, regulatory RNA, sRNA, virulence factor, SaeRS TCS, hemolysin, iron limitation, heme uptake, secretome, exoproteome

## Abstract

The Gram-positive opportunistic pathogen colonizes ∼30% of the human population but also causes various diseases. Precise regulation of genes involved in virulence and metabolic functions is required to adapt to changing host conditions, such as severe restriction of iron availability. In addition to the global regulator Fur (ferric uptake regulator), the iron limitation response of is shaped by the recently identified sRNA IsrR (iron sparing response regulator). IsrR mediates an iron sparing response by inhibiting the synthesis of nonessential iron-containing proteins, which are in particular involved in the central metabolism. In addition, we demonstrate that *isrR* expression is positively associated with α-hemolysin levels and the hemolysis activity of HG001. To investigate the influence of IsrR on virulence factor production, we performed a mass spectrometry-based secretome analysis of *isrR*-expressing and nonexpressing strains under iron-limited and iron-sufficient conditions. The SaeR regulon was positively influenced by the presence of IsrR, and IsrR is likely involved in the activation of the Sae system. Additionally, IsrR also positively affected the protein levels of the *isdABCDEFGH*-encoded heme uptake system (e.g., IsdB). Taken together, IsrR establishes a link between the iron limitation response and the virulence in .

## Graphical abstract

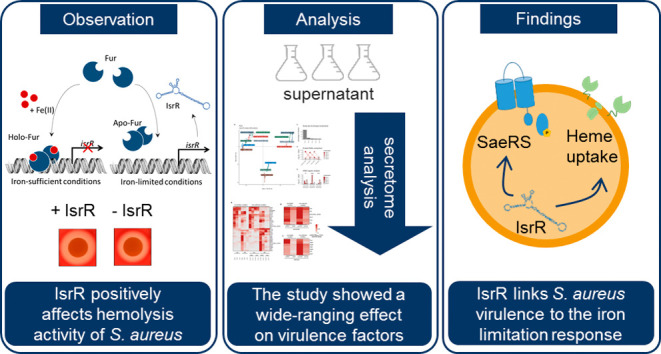

## Introduction

Virulence is a dynamic property of pathogens, which is determined by the combination of bacterial fitness traits and virulence factors directed against the host. To survive and proliferate within the host, pathogenic bacteria need to adapt their metabolism to the varying nutritional conditions encountered in different host environments.^1^ However, environmental stimuli not only trigger metabolic adaptations but also modulate the production of virulence factors.^2,3^ In line with this, various metabolic regulators influence, either directly or indirectly, the activity of virulence regulators, thereby integrating the expression of metabolic and virulence genes.^4^ Among the growth-restricting factors in mammalian hosts are the limited availability of iron and other trace metals, which are severely restricted by the host through a mechanism termed nutritional immunity.^5^ Specifically, extracellular free iron is sequestered by iron-binding proteins such as transferrin, while hemoglobin released from erythrocytes is captured by haptoglobin.^6^ It has long been known that iron serves as an environmental signal affecting the regulation of virulence determinants.^7^ The key regulator of iron homeostasis in most bacteria is the ferric uptake regulator (Fur), which mainly acts as a repressor of iron acquisition systems.^8,9^ Fur also controls an “iron sparing” response mediated by small regulatory RNAs (sRNAs).^10,11^ Moreover, Fur and Fur-regulated sRNAs play important roles in virulence-related processes in various pathogens.^1,12,13^ For instance, Fur-regulated sRNAs can also directly target virulence factor genes as shown for RyhB of^14^ and the duplicated PrrF1/PrrF2 sRNAs of .^15^

In the Gram-positive opportunistic pathogen , which can cause a wide range of diseases ranging from skin infection to sepsis,^16^ virulence is coordinated through a complex network of transcriptional and post-transcriptional regulators.^17^ As described above, this network is intertwined with regulators of central metabolism and stress responses.^1,3^ Recently, the Fur-regulated sRNA iron sparing response regulator (IsrR) of was characterized^18,19^ as a functional analogue of iron sparing response sRNAs like RyhB of^20^ and FsrA of .^21^ These sRNAs inhibit the translation of mRNAs encoding nonessential iron-containing proteins, which is associated with major changes in cellular metabolism under iron-limiting conditions.^10^ In this way, iron remains available for the essential iron-containing proteins.

In accordance with the cellular functions of iron-responsive sRNAs in other bacteria, IsrR regulates tricarboxylic acid (TCA) cycle activity,^19,22,23^ nitrate respiration,^18^ and oxidative stress response^19^ (Table 1). In addition, it has been shown that IsrR is important for the pathogenesis of infections as strains lacking IsrR exhibited decreased virulence in different mouse models (septicemia, pneumonia, and skin infection).^18,23^ However, a possible role of IsrR in the regulation of levels and activity of virulence factors has not yet been investigated. In a recent study of the global IsrR targetome,^19^ we noticed that the cellular abundance of α-hemolysin (Hla) was considerably lower in IsrR-deficient strains compared to IsrR-proficient strains. α-Hemolysin, also called α-toxin, is a pore-forming toxin known for almost 100 years^24^ that causes membrane damage^25^ and targets various host cells such as human platelets, endothelial cells, epithelial cells, and leukocytes.^26,27^ However, despite its name, it does not lyse human erythrocytes.^28^ Hla is an important virulence determinant as it is critical for the pathogenesis of in various infection models.^27^

**Table 1:** Previously Identified IsrR Targets

locus tag	gene symbol^37^	protein localization^38^	reference
Negatively Regulated by IsrR			
SAOUHSC_01347	*citB*	cytoplasmic	^19,22,23^
SAOUHSC_01802	*citZ*	cytoplasmic	^19,23^
SAOUHSC_02943	*citM*	cytoplasmic membrane	^23^
SAOUHSC_01418	*sucA*	cytoplasmic	^19^
SAOUHSC_01416	*sucB*	cytoplasmic	^19^
SAOUHSC_01327	*katA*	cytoplasmic	^19^
SAOUHSC_01103	*sdhC*	cytoplasmic membrane	^23^
SAOUHSC_01104	*sdhA*	cytoplasmic membrane	^19^
SAOUHSC_01105	*sdhB*	cytoplasmic membrane	^19^
SAOUHSC_02525	*rnd2*	cytoplasmic membrane	^19^
SAOUHSC_01846	*acsA*	cytoplasmic	^19^
SAOUHSC_02582	*fdhA*	cytoplasmic	^18,19^
SAOUHSC_01960	*hemY*	cytoplasmic	^19^
SAOUHSC_00875	*ndh2b*	cytoplasmic membrane	^19^
SAOUHSC_02409	*rocF*	cytoplasmic	^19^
SAOUHSC_01269	*miaB*	cytoplasmic	^19,39^
SAOUHSC_00198	*fadE*	cytoplasmic	^19^
SAOUHSC_02003		cytoplasmic Membrane	^19^
SAOUHSC_02760		cytoplasmic Membrane	^18,19^
SAOUHSC_01776	*hemA*	cytoplasmic	^19^
SAOUHSC_00679	*ccpE*	cytoplasmic	^19,22^
SAOUHSC_02861		cytoplasmic	^19^
SAOUHSC_03028	*bstA*	cytoplasmic	^19^
SAOUHSC_00738	*dtpT*	cytoplasmic membrane	^19^
SAOUHSC_02647	*mqo*	cytoplasmic membrane	^19,23^
SAOUHSC_02681	*narG*	cytoplasmic membrane	^18^
SAOUHSC_02684	*nasD*	cytoplasmic	^18^
Positively Regulated by IsrR			
SAOUHSC_00304		cytoplasmic	^19^
SAOUHSC_00827		cytoplasmic	^19^

Interestingly, regulation of Hla protein levels and hemolysis activity of in response to iron availability and the presence of Fur were already found by Torres *et al.* (2010).^29^ Fur also influenced the expression of additional secreted virulence factors, including the leukocidins γ-hemolysin and LukED,^29^ which are the most potent hemolytic toxins against human erythrocytes.^30^ Lysis of host erythrocytes results in the release of hemoglobin, which is an important iron source for the growth of .^31–33^ A key regulator of virulence gene expression is the two-component system SaeRS ( exoprotein expression).^34,35^ It controls the expression of approximately 40 genes in .^34^ Besides the already mentioned cytotoxin genes (*hla*, *lukED*, *hlb*, *lukGH*, and *hlgACB*), the Sae system controls additional staphylococcal virulence factor genes encoding, for example, secreted enzymes, adhesins, toxins, and immune evasion proteins.^34^

As most *bona fide* virulence factors are extracellular proteins or cell wall-anchored proteins,^17,36^ we performed a secretome analysis under iron-sufficient and iron-limited conditions to study the impact of IsrR on virulence factor expression in . We introduced a normalization method to analyze protein abundances in culture supernatants, which allowed precise calculation of the ratios of the actual protein amounts between the respective samples even if the sample concentrations were very different. Our results demonstrate that IsrR positively affects the protein levels of several secreted virulence factors as well as the hemolysis activity of . These effects are most likely based on the IsrR-driven activation of the Sae system. In addition, the secretome analysis revealed that IsrR was associated with increased protein abundance of the Isd system responsible for heme uptake. IsrR thus provides a link between virulence and the iron limitation response, which are both critical for survival and pathogenicity of during infection processes.

## Experimental Section

### Bacterial Strains

Bacterial strains and plasmids used in this study are given in Table 2.

**Table 2:** Bacterial Strains and Plasmids Used

*S. aureus*	relevant genotype/characteristics	reference
Strains		
HG001	*rsbU*^+^-repaired and *tcaR*-defective derivate of NCTC 8325	^40^
SGB007	HG001 Δ*isrR*::*ermB*, P_*isrR*_ Shine–Dalgarno sequence *pgi* ^a^	^19^
SGB009	HG001 Δ*fur*::*ermC*, P_*fur*_ Shine–Dalgarno sequence *fur* ^a^	^19^
SGB010	HG001 Δ*fur*::*ermC*, P_*fur*_ Shine–Dalgarno sequence *fur* ^a^ and Δ*isrR*::*ermB*, P_*isrR*_ Shine–Dalgarno sequence *pgi* ^a^	^19^
SGB011	HG001 pJLisrR	^19^
SGB012	HG001 pJLctrl	^19^
SGB013	HG001 Δ*saePQRS*	this study
SGB014	HG001 Δ*saePQRS* pJLisrR	this study
SGB015	HG001 Δ*saePQRS* pJLctrl	this study
SGB016	HG001 Δ*hla*::*ermC*	this study
Plasmids		
pJL-sar-isrR (pJLisrR)	pJL-sar plasmid with *isrR* of HG001 under the control of constitutive promoter P_*sarA*P1_ of *S. aureus* RN6734	^19^
pJL-sar-ctrl (pJLctrl)	pJL-sar plasmid with the constitutive promoter P_*sarA*P1_ of *S. aureus* RN6734 directly followed by the terminator (control with no gene of interest under the control of P_*sarA*P1_)	^19^

a Expression of the *ermC* or *ermB* resistance gene is controlled by the promoter and Shine–Dalgarno sequences indicated.

The markerless *saePQRS* deletion mutant in HG001 background, named SGB013, was obtained by transforming HG001 with the mutagenesis vector pCG335.^41^ Mutagenesis was performed as described in Bae and Schneewind (2006).^42^ Deletions were verified by PCR and were phenotypically. Plasmids isolated from the HG001 strains SGB011 and SGB012 were used for the transformation of strain SGB013 (HG001 Δ*sae*) *via* electroporation according to Augustin and Götz (1990).^43^ Plasmid sequences were validated *via* Sanger sequencing. The *hla* allele exchange mutant SGB016 was generated by transduction of the Δ*hla*:*:ermC* allele from DU190 ^44^ *via* φ11.

### Media and Growth Conditions

 strains were in general cultivated as described by Ganske *et al.* (2024).^19^ Strains were cultivated in a tryptic soy broth (TSB; BD, USA) and an iron-depleted TSB medium (TSBDP). For iron depletion, the iron chelator 2,2′-dipyridyl (DP) was added to the medium at a concentration of 600 μM, followed by incubation for at least 1 h at 37 °C. The main cultures were grown aerobically by orbital shaking at 220 rpm at 37 °C after inoculation with an exponentially growing TSB preculture at an optical density at 540 nm (OD_540nm_) of 0.05 in an Innova 44 (New Brunswick Scientific, USA) incubator. The culture medium of plasmid-carrying strains was supplemented with 10 μg/mL chloramphenicol.

For anaerobic cultivation, HG001 and HG001 Δ*isrR* were initially cultivated aerobically until an OD_540nm_ of 1. Then, to accomplish anaerobic cultivation conditions, the culture volume was transferred into 15 mL centrifugation tubes with screw lids for samples to be harvested for further molecular analyses and, additionally, in 2 mL storage tubes with screw lids as aliquots for OD_540nm_ measurements. Tubes were completely filled, and lids were screwed. Samples were harvested 8 h after initial inoculation of the main culture. As aerobic control, culture volume was also transferred into a new flask filled to 20% of the flask volume. All split cultures were then incubated at 37 °C with shaking.

### Blood Agar Hemolysis Assay

Bacterial strains were cultivated in TSB as described above. At an OD_540nm_ of 1 (exponential growth phase), for each sample, 10 μL of culture was dropped on Columbia blood agar plates containing 5% of sheep blood. Plates were incubated for 24 or 48 h, as indicated for the respective data. Hemolysis activity was quantified as halo area in mm^2^ around the bacterial cell spot.

### RNA Preparation and Northern Blot Analysis

The bacterial strains were cultivated as described above. In the exponential and stationary growth phase, bacterial cells were harvested 2.5 and 6 h after inoculation of the main culture. About 15 OD_540nm_ units were harvested with 1/3 volume of frozen killing buffer (20 mM Tris/HCl [pH 7.5], 5 mM MgCl_2_, and 20 mM NaN_3_) by subsequent centrifugation for 3 min at 10,000*g* and 4 °C. After discarding the supernatant, cell pellets were frozen in liquid nitrogen and stored at −70 °C. Subsequent mechanical bacterial cell disruption and RNA preparation were carried out as described previously.^45^ For each sample, 4 μg of total RNA was used for Northern blot analysis. Northern blotting was performed as recently described by Ganske *et al.* (2024).^19^ Probes and respective primers are listed in Table S1.

### Harvest and Preparation of Supernatant Samples for Mass Spectrometric Analysis

Strains were grown as described above. In the exponential growth phase (2.5 h after the inoculation of the main culture) and in the stationary growth phase (8 h after the inoculation of the main culture), supernatant samples were harvested. For each sampling point, 10 mL of culture was taken, immediately cooled down in liquid nitrogen to inactivate proteases, and subsequently centrifuged (3 min, 10,000*g*, 4 °C) to remove cells from the medium. Each 8 mL of the upper supernatant was carefully transferred into a new reaction tube, frozen in liquid nitrogen, and stored at −70 °C.

Prior to protein concentration determination, proteins of the supernatant samples were precipitated for 48 h at 4 °C using a final concentration of 15% (v/v) trichloroacetic acid (TCA). Then, precipitated proteins were pelleted by centrifugation (1 h, 17,000*g*, 4 °C). Protein pellets were washed multiple times with 70% (v/v) precooled ethanol and subsequently centrifuged (10 min, 17,000*g*, 4 °C) until the pellet was colorless, followed by a final 100% (v/v) ethanol washing step and centrifugation (10 min, 17,000*g*, 4 °C). The ethanol was discarded, and the pellet was dried for 30 min at room temperature (RT). Finally, the pellet was suspended in 20 mM 2-[4-(2-hydroxyethyl)­piperazin-1-yl]­ethanesulfonic acid (HEPES; pH 8.0) and stored at −70 °C. Protein concentration was determined using a Bradford assay (Biorad, Germany).

To ensure robust and reliable relative quantification between highly variable supernatant samples, a heavy ^15^N isotope-labeled standard was added to each nonprecipitated supernatant sample. The amount added corresponded to 20% of the mean supernatant protein concentration across all samples. This amount was used to ensure a sufficient amount of standard protein added to the high-concentrated stationary growth phase samples for reliable detection and quantification of the standard and, at the same time, a sufficient low amount of standard protein added to the low-concentrated exponential growth phase samples for reliable detection and quantification of the sample.

This approach resulted in theoretically approximately 50% standard in exponential growth phase samples and theoretically approximately 10% standard in stationary growth phase samples. Generation of the external heavy ^15^N isotope-labeled standard is described in detail in Supporting Information Method S1. Supernatant protein with supplemented standard of each sample was precipitated using the TCA method as described above, and protein concentrations were determined again using the Bradford assay (Biorad, Germany).

Tryptic digestion of proteins and peptide purification were performed as described in Blankenburg *et al.* (2019)^46^ and Reder *et al.* (2024)^47^ with minor adjustments: 3.2 μg of protein sample was mixed with 3.2 μL of hydrophilic (GE Healthcare, Little Chalfont, UK) and hydrophobic (Thermo Fisher Scientific, MA, USA) carboxylate-modified magnetic SeraMag Speed Beads (1:1; 20 μg/μL) and acetonitrile (ACN) to a final concentration of 80% (v/v), and samples were incubated for 5 min at RT with shaking. The beads with bound proteins were washed twice with 80% (v/v) ethanol and once with 100% (v/v) ACN on a magnetic rack. For protein digestion, the beads were rebuffered into 50 mM Tris–HCl 1 mM CaCl_2_ (pH 8.0) and incubated with 130 ng of Trypsin/LysC Mix (Promega, Madison, USA) for 16.5 h at 37 °C. The digestion was stopped, and peptides were eluted by addition of 0.5% (v/v) trifluoracetic acid. After two steps of centrifugation (1 min, 17,000*g*, RT) and incubation at the magnetic rack, peptide samples were fully separated from the beads and transferred to an MS vial. Peptide concentration was determined using a Quantitative Peptide Assay & Standards (Pierce, Thermo Fisher Scientific, MA, USA).

### Mass Spectrometric Measurements and Data Analysis

For nanoLC-MS/MS analysis, tryptic peptides were separated on an Ultimate 3000 nano-LC system (Thermo Fisher Scientific, USA) and subsequently analyzed on an Orbitrap Exploris 480 mass spectrometer (Thermo Fisher Scientific, USA) in data-independent acquisition (DIA) mode, and injection volume was adjusted based on the peptide concentration of the sample. Summarized details of the MS/MS analysis are presented in Tables S2 and S3.

DIA MS data was analyzed using a spectral library-based approach in the Spectronaut software (version 18.6.231227.55695; Biognosys AG, Switzerland). A dedicated spectral library was built for this project, comprising DIA- and DDA-MS measurements of NCTC 8325 lineage strains under several stress and infection conditions (106,906 precursors).

Database searches were performed against a NCTC 8325 protein database (*Aureo*Wiki^37^) where the RsbU sequence was replaced by the Newman RsbU sequence to represent the HG001 protein sequence (2853 staphylococcal protein sequences, four marker protein sequences, and four contaminant protein sequences).

For normalization, a second spectral library integrating measurements of the complex heavy-labeled standard (14,768 precursors) was created by searching against a 168 protein database (4201 protein sequences).

Detailed parameters for the search and library construction are summarized in Table S4. Ion intensities were global median normalized based on only the heavy-labeled ions identified in all samples to allow robust and reliable relative quantification of the staphylococcal supernatant proteins.

The mass spectrometry proteomics data, corresponding protein databases, and spectral libraries have been deposited to the ProteomeXchange Consortium *via* the PRIDE partner^48^ repository with the data set identifier PXD055092.

Based on identified ion counts and normalization factors, one outlier sample was manually excluded from the data set. Heavy-labeled standard proteins were excluded from the data set prior to subsequent statistical analyses. The secretome data was analyzed in R (v4.4.1) using a specifically project-adjusted version of the SpectroPipeR pipeline^49^ for downstream data processing and R packages listed in Table S5.

For further analysis, peptide ions of proteins of interest for the secretome were selected, methionine-oxidized peptides were excluded from the data set, and only proteins identified with at least two peptides were considered. Proteins of interest were identified based on the following criteria: (i) protein localization was predicted as extracellular, associated with the cell wall or the cell membrane according to DeepLocPro^38^ or (ii) PSORTb,^50^ (iii) a signal peptide was predicted based on SignalP,^51^ or (iv) the relative iBAQ value^52^ was at least 2-fold higher in the secretome data in exponential or stationary growth phase compared to the cellular proteome recorded by Ganske *et al.* (2024).^19^ The complete classification of annotated proteins of HG001 as proteins of interest can be found in Table S9. Localization of proteins was predicted using DeepLocPro v1.0 ^38^ with “group positive”. PSORTb^50^ based localization prediction and SignalP^51^ based signal peptide prediction were obtained from *Aureo*Wiki.^37^

Peptide ions with a *q*-value less than 0.001 and identification in at least 50% of one condition were selected for analysis. Peptide intensities were calculated as the sum of corresponding ion intensities, and the peptide intensities were subsequently condition-wise median–median normalized to reduce noise introduced by manual addition of the heavy-labeled standard. Based on the normalized peptide intensities, maxLFQ values^53^ were calculated and further condition-wise median–median normalized. Relative iBAQ values were calculated as the percentage of the respective Spectronaut-based iBAQ intensity^52^ of the total sum of iBAQ intensity per condition. Statistics for condition-wise comparison of protein abundance was calculated using the ROPECA approach^54^ with subsequent Benjamini–Hochberg *p*-value adjustment.^55^

The two label-free protein quantification methods used in this work are explained in brief: iBAQ^52^ (intensity-Based Absolute Quantification) estimates the relative abundance of proteins within a sample. The iBAQ intensity for each protein is calculated by dividing the sum of peptide intensities of the protein by the number of theoretically observable tryptic digested peptides for that protein. MaxLFQ^53^ (Maximal Peptide Ratio Extraction and Label-Free Quantification) estimates protein abundances using peptide intensity ratios between samples. The maximum available peptide ratios of all peptides that belong to a protein are taken per sample pair, and pairwise protein ratios aggregated as the median of the peptide ratios are used to solve the resulting system of linear equations to estimate the protein abundance in each sample. This results in an accurate abundance profile for each protein across all of the samples.

For prediction of NCTC 8325 (RefSeq-Accession: NC_007795) strain-specific IsrR targets, IntaRNA2.0 v3.3.1 ^56^ was used with RNA web tools v5.0.10 ^57^ and Vienna RNA packages v2.5.0.^58^ Genome-wide targets were predicted at the 5′-end 200 bp upstream to 100 bp downstream of the respective start codon.

### Siderophore Activity Assay

Bacterial strains were cultivated as described above. In the stationary growth phase, samples were harvested by filtration (pore size, 0.45 μm). The filtered supernatant was stored at −20 °C. Siderophore activity was quantified using the Chrome azurol S (CAS) agar diffusion assay^59^ based on the CAS assay.^60^ CAS agar was prepared as described in Shin *et al.* (2001),^59^ and exactly 25 mL of CAS agar was aliquoted in Petri dishes. Holes were punched into the gel by using a 4 mm biopsy puncher. For each sample, 30 μL of supernatant was filled into one hole and after 2 h of incubation at 37 °C, additional 30 μL was added per sample. Plates were incubated for a total of 5.5 h. Medium controls were used as standards between plates. Diameters of orange-colored diffusion halos were measured, and halo areas were normalized to the respective cell density in OD_540nm_ at the harvest time point.

## Results

### IsrR Activates *hla* Expression in an Indirect Manner

In our recent work,^19^ the global IsrR targetome was characterized by combining proteomics-based experimental identification of potential IsrR targets and *in silico* target prediction using CopraRNA2.^61^ Two experimental approaches were pursued. First, proteome profiles of HG001 were compared with those of the isogenic Δ*isrR* mutant SGB007 under iron-limited growth conditions (TSB + 600 μM DP), where Fur-dependent repression of *isrR* is relieved. In the second approach, the effects of IsrR on the bacterial proteome were analyzed under iron-replete conditions (TSB) using a strain in which *isrR* is constitutively expressed from a plasmid under the control of the *sarA* P1 promoter. Among the proteins that showed the strongest dependence on IsrR in both experimental approaches was α-hemolysin Hla, whose levels were approximately 9-fold higher in the *isrR*-expressing strains (stationary phase; Figure 1A). However, no interaction between IsrR and *hla* mRNA was predicted in CopraRNA2-based target prediction.^19^ In addition, analysis of the sole NCTC 8325 reference genome using IntaRNA2.0,^56^ which detected additional strain-specific targets, could also not reveal any interaction between IsrR and *hla* mRNA (sequence range 200 bp upstream to 100 bp downstream of the start codon and 0.05 *p*-value cutoff; Table S7).

**Figure 1:**
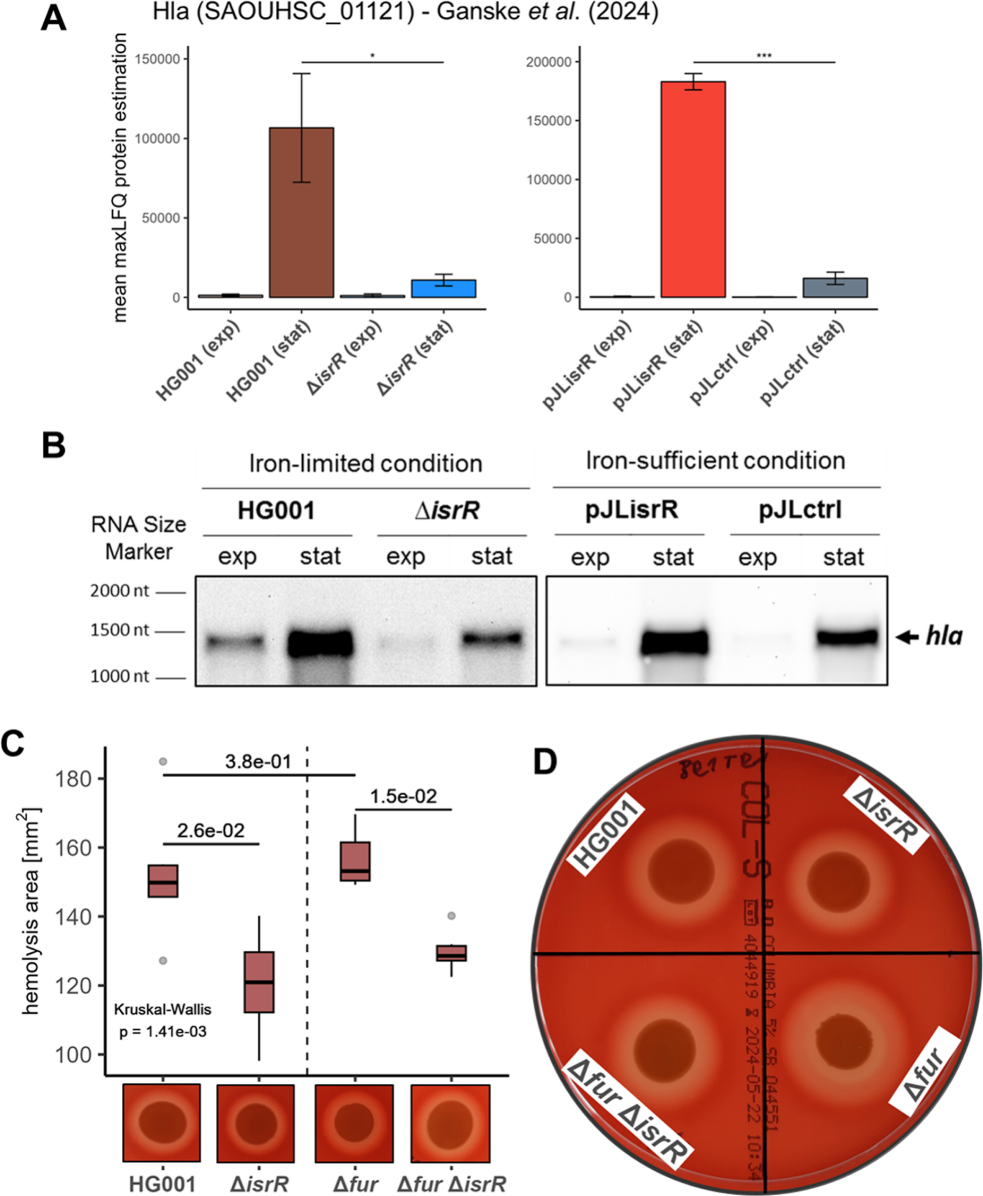
Hla protein levels, *hla* transcript levels, and hemolysis activity are influenced by IsrR. (A) Protein levels of Hla according to Ganske *et al.* (2024).^19^ The bar chart depicts the amount (mean maxLFQ protein level) of Hla between *isrR*-expressing (HG001, pJLisrR) and nonexpressing strains (Δ*isrR*, pJLctrl). Error bars represent the standard deviation of the four biological replicates. Statistics: Welch-*t* test on protein levels (*p* < 0.001: ***, *p* < 0.01: **, and *p* < 0.05: *). (B) The effect of IsrR on *hla* mRNA abundance was examined by Northern blot analysis. For each sample, 4 μg of total RNA was loaded per lane. (C) The effect of IsrR on hemolysis activity. Strains were cultivated in TSB at 37 °C and at an OD_540nm_ of 1, 10 μL of culture was spotted on 5% sheep blood Columbia agar, and plates were incubated for 24 h. Hemolysis activity was determined based on the area of hemolysis around the spot of growing cells. Boxplots represent the median of three biological and two technical replicates each. Statistics: Kruskal–Wallis test on hemolysis activity and Wilcoxon test with Benjamini–Hochberg *p*-value adjustment as post hoc test. Results of relevant post hoc pairwise comparisons are depicted. (D) Hemolysis activity after 48 h. Strains were cultivated in TSB at 37 °C and at an OD_540nm_ of 1, 10 μL of culture was spotted on 5% sheep blood Columbia agar, and plates were incubated for 48 h. Hemolysis activity was determined based on the area of hemolysis around the spot of growing cells.

In order to investigate whether IsrR-dependent regulation of *hla* takes place on the transcriptional level, Northern blot analysis was carried out using the same strains and growth conditions as those for the proteome analysis (Figure 1B). Indeed, in accordance with the Hla protein levels, *hla* transcript levels were decreased in the non-*isrR*-expressing strains HG001 Δ*isrR* under iron-limited conditions and HG001 pJLctrl under iron-replete conditions compared to the respective *isrR*-expressing strains HG001 and HG001 pJLisrR. Northern blot analysis confirmed that *hla* is mainly expressed in the stationary phase. Since virulence factor gene expression^62^ and in particular *hla* expression^63^ are oxygen-status dependent, we explored if a similar expression pattern was also observed under conditions of oxygen limitation, which is a known feature of the host environment.^1,64–66^ We could demonstrate that the decrease in the *hla* transcript in the Δ*isrR* mutant compared to the HG001 wild-type was also observable under infection-mimicking conditions of iron and oxygen limitation, albeit at much lower total *hla* mRNA levels (Figure S1).

### Hemolytic Activity of Is Enhanced by IsrR

Next, we wanted to analyze Hla activity by quantification of the staphylococcal β-hemolysis activity on Columbia agar plates containing 5% sheep blood.^29,44^ The involvement of the α-, β-, γ-, and δ-hemolysins in hemolysis varies between the blood donor organisms and staphylococcal strains.^44,67^ α-Hemolysin very efficiently lyses rabbit erythrocytes,^24,44^ whereas human erythrocytes are only weakly lysed by it.^68^ In particular, HG001 lyses sheep erythrocytes on agar plates with the help of α- and δ-hemolysin,^40^ as the β-hemolysin gene is disrupted by the integration of the prophage φ13 ^69^ and γ-hemolysin is inhibited by agar.^70^ Analysis of hemolysis activity of a HG001 Δ*hla* mutant revealed negligible hemolysis activity of this strain on sheep blood agar (Figure S2A). Hemolysis activity of the HG001 strain can thus be used as a readout for Hla activity. To assess the influence of IsrR on hemolysis activity, the strains HG001, HG001 Δ*isrR*, HG001 Δ*fur*, and HG001 Δ*fur*Δ*isrR* were analyzed (Figure 1C,D). Hemolysis activity was significantly reduced for the Δ*isrR* mutant compared to the HG001 wild-type as well as for the Δ*fur*Δ*isrR* mutant compared to the Δ*fur* mutant, demonstrating that IsrR enhances the hemolytic activity of . The Δ*fur* mutant strain, constitutively expressing *isrR*,^18,19^ exhibited slightly higher hemolysis activity than the HG001 wild-type after 24 h of incubation, which increased to a 1.4-fold difference after 48 h of incubation (Figures 1D and S2C). This was in line with previous studies of Torres *et al.* (2010)^29^ and Schmitt *et al.* (2012)^67^ where Δ*fur* mutant strains exhibited higher hemolysis activity than the respective wild-type strains.

### Effect of IsrR on Hemolytic Activity and Virulence Factor Expression Is Linked to the Activity of the Sae System

Multiple regulators coordinately control the expression of *hla*.^17,71^ However, the *hla* expression essentially depends on transcriptional activation by the SaeRS two-component system.^67,72,73^ In line with this, the hemolysis activity of a HG001 Δ*saePQRS* mutant was comparable to the activity of the Δ*hla* mutant strain (Figure S2A). In the Sae regulatory system, SaeS is the membrane-bound sensor histidine kinase activating the cytoplasmic DNA-binding response regulator SaeR.^35^ Binding of phosphorylated SaeR activates transcription of target genes such as *hla*.^74,75^ The accessory proteins, the lipoprotein SaeP and the membrane protein SaeQ, modulate the phosphatase activity of SaeS.^76,77^

As described above and shown in Figure S2A, the hemolytic activity of the Δ*saePQRS* mutant was significantly reduced compared to the activity of the HG001 wild-type. The extent of the reduction was greater than for the *isrR* deletion mutant (Figures 1C and S2B). In contrast to the case for the wild-type, introduction of the *isrR*-expression plasmid pJLisrR into the Δ*sae* mutant did not increase its hemolytic activity (Figure 2A). These results indicated that the observed effect of IsrR on *hla* expression, Hla protein abundance, and hemolysis activity requires the activation of *hla* transcription by SaeR.

**Figure 2:**
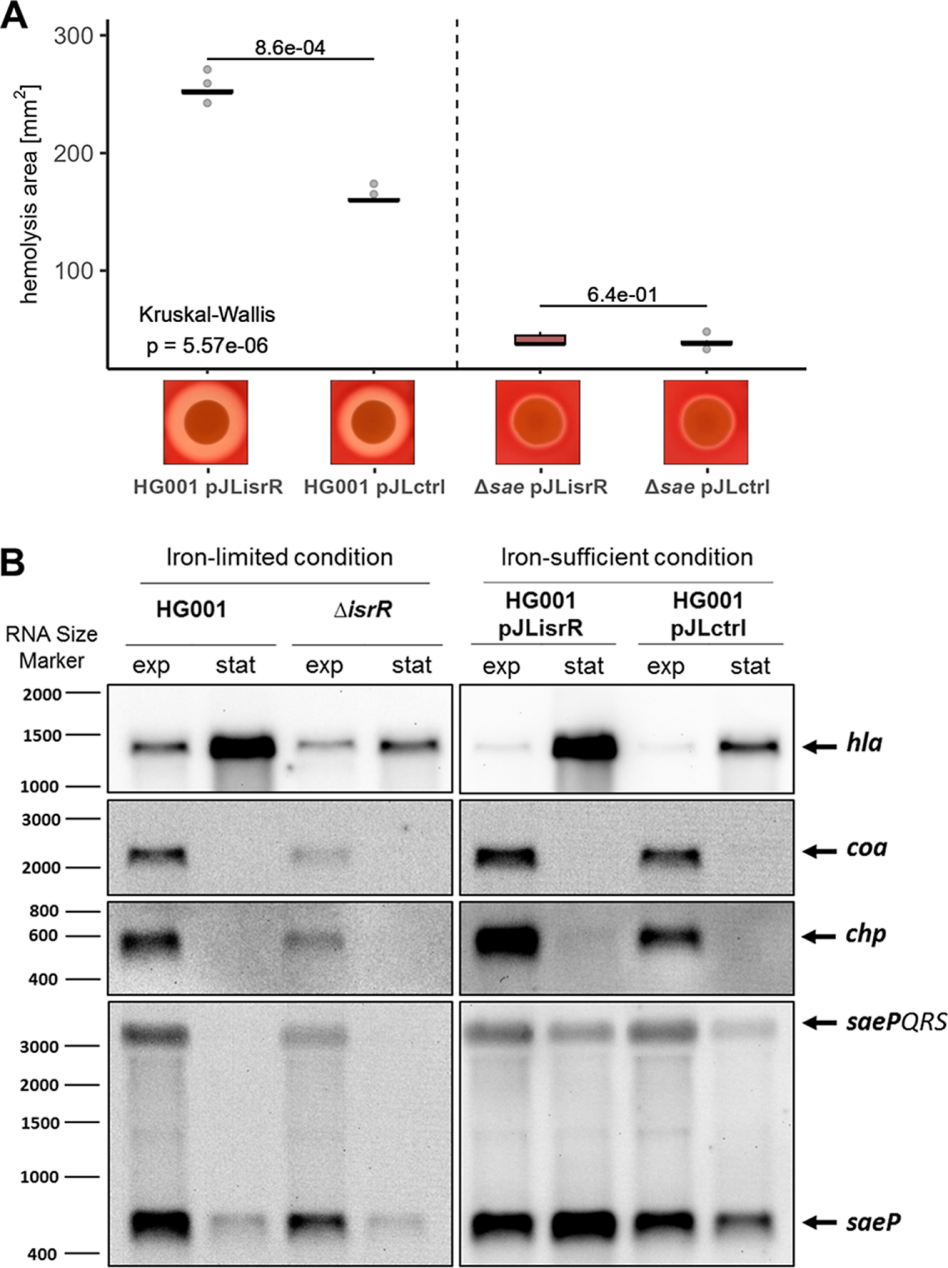
The effect of IsrR on Hla is SaeRS-dependent. (A) The effect of SaeRS and IsrR on the hemolysis activity. Strains were cultivated in TSB and at an OD_540nm_ of 1, and 10 μL of culture was spotted on 5% sheep blood Columbia agar. Plates were incubated for 24 h. Hemolysis activity was determined based on the area of hemolysis around the spot of growing cells. Boxplots represent the median of three biological and two technical replicates each. Statistics: Kruskal–Wallis test on hemolysis activity and Wilcoxon test with Benjamini–Hochberg *p*-value adjustment as post hoc test. Results of relevant post hoc pairwise comparisons are depicted. (B) The effect of IsrR on *hla*, *chp*, *coa*, and *saePQRS* mRNA abundance representing SaeR targets was examined by Northern blot analysis. For each sample, 4 μg of total RNA was loaded per lane.

To investigate if the observed IsrR-driven effect on Hla is associated with the activity of the Sae system, the transcript levels of three other SaeR-regulated genes, *coa*, *chp*, and *saeP*,^34,35,78^ were analyzed with regard to the influence of IsrR. The *saePQRS* operon is mainly transcribed from the two promoters P1 and P3.^73,79–81^ The P1 promoter located upstream of the complete operon has the highest activity and is strongly autoregulated by binding of SaeR.^73,74,79,82^ Northern blot analysis under *isrR*-expressing and nonexpressing conditions, as with the investigation of the *hla* transcript levels (Figure 1B), revealed a positive effect of *isrR* expression on transcript levels of *coa*, *chp*, and *saeP* (Figure 2B).

In agreement with this result, reassessment of the data of the cellular proteome^19^ showed that the protein levels of SaeR regulon members were also generally increased in *isrR*-expressing conditions (Figure S3), even though most are secreted proteins which are not specifically covered by the analysis of the cellular proteome. The SaeR regulon was enriched in the group of proteins exhibiting higher abundance in *isrR*-expressing strains compared to nonexpressing strains (Gene set enrichment analysis (GSEA)^83^: *p*-value for exponential growth phase = 0.01 and *p*-value for stationary growth phase = 0.0011). The protein levels of SaeR and SaeS were mildly changed, and SaeP was altered in the same way as the other SaeR regulon members (Figure S4), reflecting autoregulation of the *saePQRS* operon. We then checked whether the mRNAs encoding the Sae proteins are potential targets of IsrR. None of these mRNAs were predicted as IsrR targets in the previous analysis by Ganske *et al.* (2024)^19^ or in the NCTC 8325-specific reanalysis (Table S7). Furthermore, it cannot be assumed that changes in the protein levels of SaeR and SaeS would have an impact on SaeR activity as *saeRS* overexpression has no effect on the expression of the SaeR regulon.^84^ Therefore, we conclude that IsrR modulates SaeRS activity but not protein levels of the TCS.

### Analysis of the Impact of IsrR on the Secretome

Most classical virulence factors are secreted or associated with the cell surface, enabling direct interaction with the host,^17,40^ and thus, we aimed to analyze the secretome of *isrR*-expressing and nonexpressing strains under iron-limited and iron-sufficient conditions and in the presence or absence of the SaeRS TCS to gain insight into the IsrR-driven regulation of virulence factor expression (Figure 3A).

**Figure 3:**
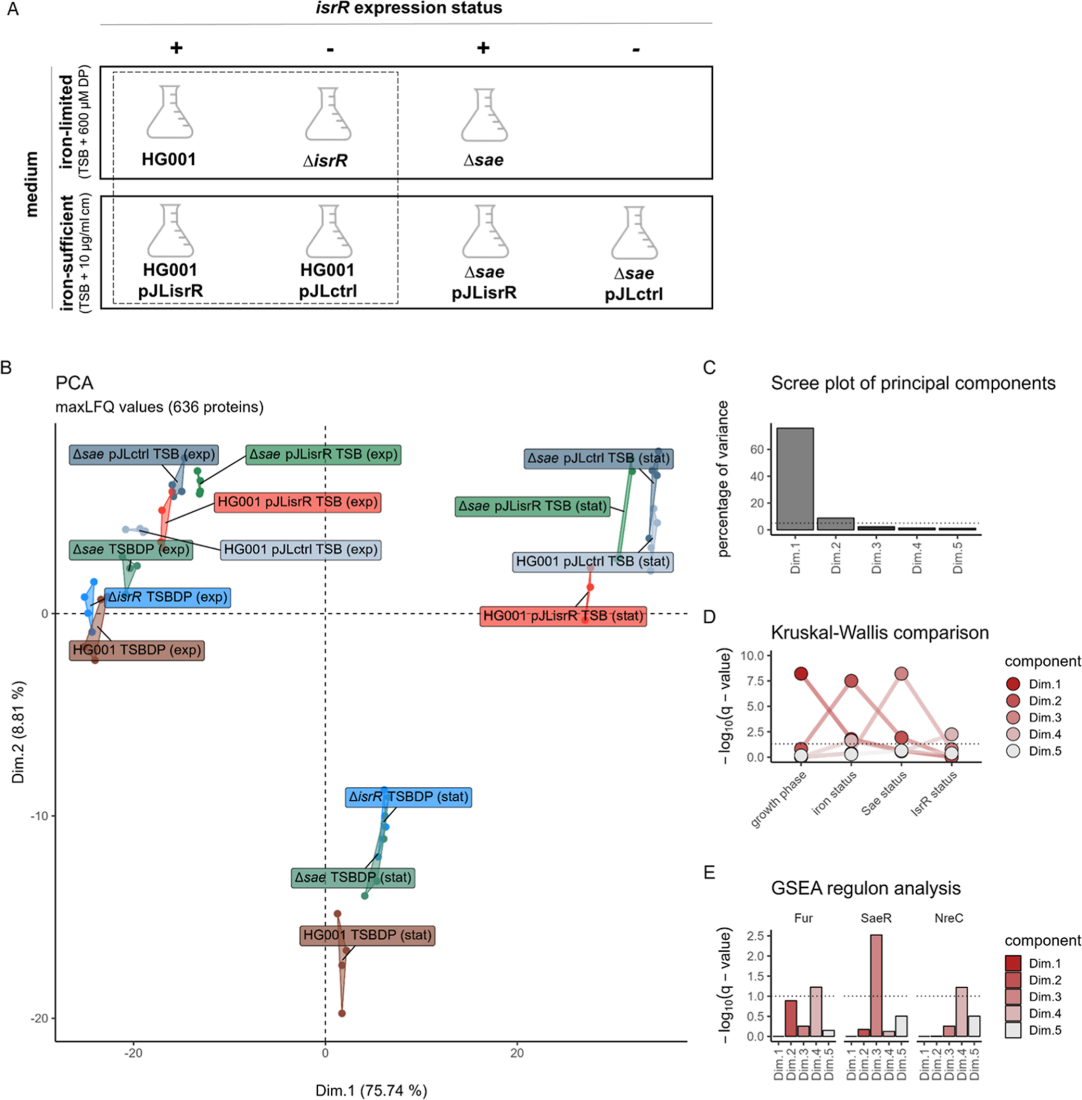
Overview of secretome profiles. (A) Schema of the experimental set up of the secretome analysis. The HG001 wild-type, the Δ*isrR* mutant, and the Δ*saePQRS* (Δ*sae*) mutant were cultivated under iron-limited conditions. Strains constitutively expressing *isrR* (pJLisrR) and control strains with the empty vector (pJLctrl) were cultivated under iron-rich conditions. The strains marked by the box were introduced in Ganske *et al.* (2024).^19^ (B) PCA displaying the first and second component. The PCA was calculated based on the 636 proteins of interest for the secretome. Each strain and sampling condition was labeled individually. Replicates are displayed as points. (C) Scree plot of principal components. The percentage of global variance described by the respective component is displayed. Components describing more than 5% of variance are colored in light gray, and components describing more than 1% of variance are displayed. The 5% threshold is depicted as the dotted line. (D) Separation profiles for the principal components. Negative decadal logarithms of *q*-values (FDR-adjusted *p*-values) of Kruskal–Wallis tests for separation of each condition subcategory (growth phase, medium, Sae status, and IsrR status) for the most important five principal components are displayed. The *q*-value threshold for a significance of 0.05 is depicted as the dotted line. (E) GSEA regulon analysis of proteins spanning the principal components. One-sided GSEA analysis was performed on the percentage of weight of each protein into each principal component for transcription factor regulons according to *Aureo*Wiki.^37^ Negative decadal logarithms of *q*-values (FDR-adjusted *p*-values) are displayed for the five most important principal components. Regulons with a *q*-value less than 0.1 (dotted line) for at least one principal component are shown, and only regulons with more than five identified members were considered.

By sampling of the culture supernatant (Figure S5) and subsequent precipitation of contained proteins, we significantly enriched extracellular proteins and proteins associated with the cell surface based on their mean relative iBAQ intensities, while we depleted the membrane and cytoplasmic proteins compared to the cellular proteome samples presented in Ganske *et al.* (2024)^19^ (Figures S6 and S7). Protein localization (Table S8) was predicted using DeepLocPro.^38^ Strikingly, we observed that some proteins predicted to be located in the cytoplasm exhibited higher relative abundance in the supernatant fraction, which might occur by nonclassical secretion mechanisms^85,86^ or false predictions. Based on relative iBAQ values (Figure S8), the secretome is dominated by IsaA during the exponential growth phase (7.2%–10.3%). IsaA is known to be one of the most abundant exoproteins of .^87,88^ Further, in line with our previous observations, during the stationary phase, Hla was with 3.6% and 3.3%, one of the most abundant proteins in SaeRS-positive and *isrR*-expressing strains (i.e., HG001 pJLIsrR under iron-rich and HG001 under iron-limited conditions). In addition, under iron limitation in the stationary phase, the Fur-regulated heme-binding protein IsdA^89–91^ accounted for 4.3–10.2% of the overall secretome fraction.

In the total secretome analysis, 1420 proteins were identified with at least two peptides. For further analysis, we aimed to remove the data of cellular proteins present in the supernatant due to cell lysis to ensure a more reliable analysis of the secretome. For that, we used predictions of protein localization, which resulted in 1232 “proteins of interest” for the secretome analysis (Experimental Section and Table S9). Of these, 394 proteins were identified in our analysis. Furthermore, to account for potentially incorrect prediction and nonclassical secretion mechanisms, we compared relative protein abundances of the secretome and the cellular proteome. 407 proteins exhibited 2-fold higher abundance in the secretome compared to the cellular proteome, of which 242 were predicted cytosolic proteins. These 242 proteins were included in the group of “proteins of interest” for the secretome (Experimental Section and Figure S6). In total, this resulted in 636 proteins for further analysis, whereas proteins not belonging to the “proteins of interest” were removed from the data set.

For subsequent maxLFQ-based protein level estimations and statistical analysis, peptide ion intensities were normalized to an external standard of fixed amount for all samples (Experimental Section and Figure S9), which allowed more precise calculation of the ratios of the actual protein amounts between the respective supernatant samples. A principal component analysis (PCA; Figure 3B–E) revealed that ∼90% of the secretome profile variance could be explained by the five first components (Figure 3C). The first component, Dim.1 (Figure 3B), explained 75.74% of the total variance and significantly separated the secretome profiles according to the respective growth phase and to a lesser extent according to the growth medium, which represents the iron status (Figure 3D). The second component, Dim.2 (Figure 3B), reflected the expected differences in the secretome profiles caused by the iron-limited and iron-sufficient medium, which were mainly driven by proteins belonging to the Fur regulon (Figure 3E). The further components Dim.3 and Dim.4 reflected the differences in the secretome profiles in terms of the Sae and IsrR status (Figure 3D), which were driven by proteins associated with the SaeR regulon and the NreC and Fur regulons, respectively. The NreC regulon contains the known IsrR targets *narG* and *nasD*.^18^

Based on the PCA, we conclude that the secretome profiles are specific with regard to the growth phase, iron availability, *isrR* expression status, and the presence of the Sae system.

### SaeR-Dependent Virulence Factors Are Affected by IsrR

Next, we analyzed the effect of the IsrR status of cells on the protein abundance of SaeR-regulated virulence factors (Table S6), which are secreted or cell surface-associated proteins^34,92,93^ (Figure 4). To verify the SaeR-dependent regulation of known regulon members under the conditions of our study, protein levels of the Sae-negative strains were included in the analysis. Finally, to take into account that the amount of secreted proteins also depends on the cell number, we normalized the maxLFQ protein levels to the OD_540nm_ of the corresponding sample to ensure comparability between the growth phases and the cultivations with different iron availabilities.

**Figure 4:**
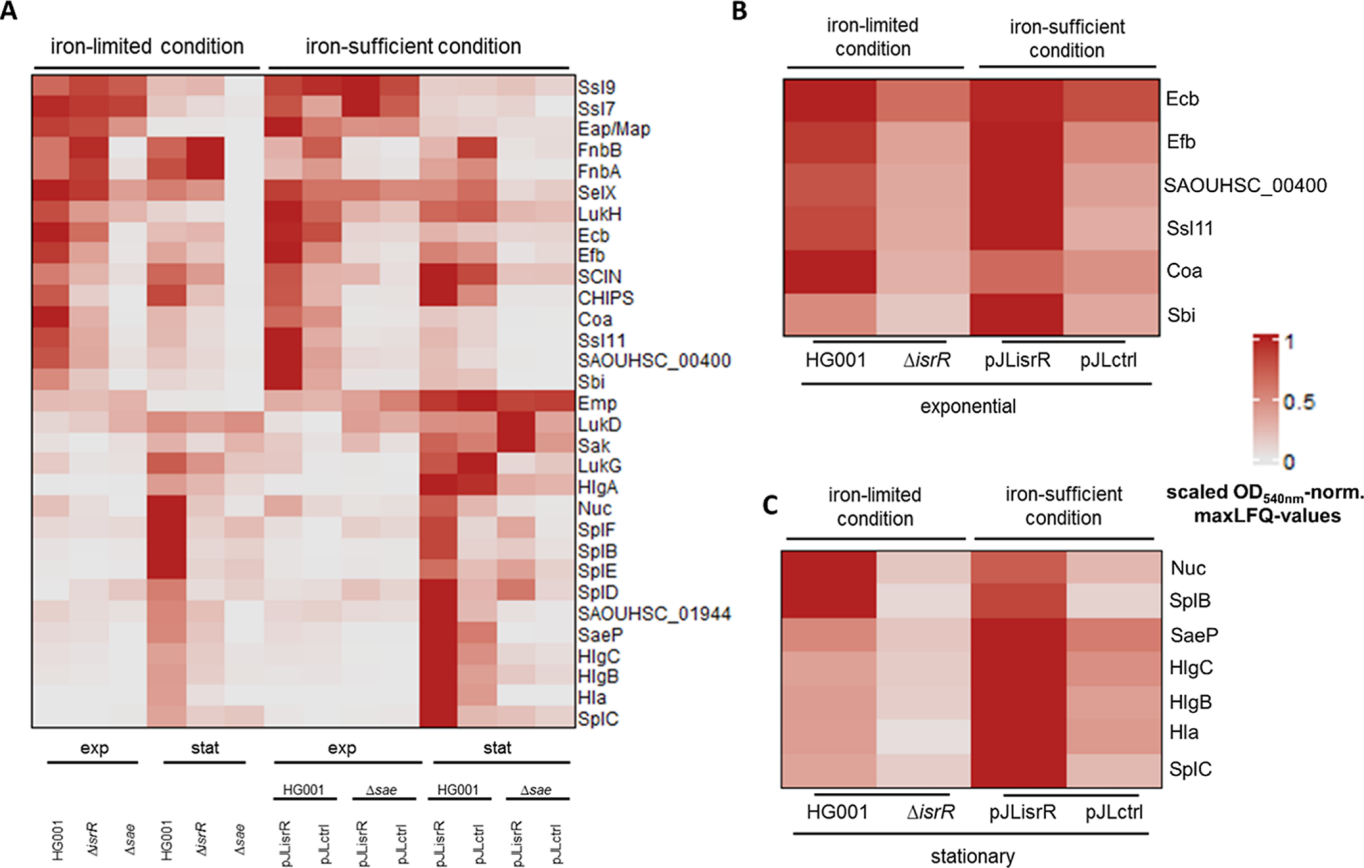
Heat map of protein abundances of known Sae-dependent virulence factors in the secretome. MaxLFQ values were normalized to the mean OD_540nm_ per condition at the harvest time of the supernatant sample and min–max scaled per protein across iron-sufficient and iron-limited conditions. The SaeR regulon was depicted according to *Aureo*Wiki. (A) Global representation of protein levels in the secretome of the known SaeR regulon. (B) Strictly SaeR-dependent regulon members with higher protein levels during the exponential growth phase are depicted for the *isrR*-expressing and nonexpression Sae-positive strains. (C) Strictly SaeR-dependent regulon members with higher protein levels during the stationary growth phase are depicted for the *isrR*-expressing and nonexpression Sae-positive strains.

The data in Figure 4 show a strong growth phase dependency of the SaeR-dependent proteins in the secretome, which is in line with previous studies.^92,94^ Among the virulence factors with higher levels during the exponential growth phase were Ssl11, Ecb, Efb, Sbi, and Coa (Figure 4B), whereas, e.g., SplB, SplC, HlgC, HlgB, Nuc, and Hla showed higher levels during the stationary growth phase (Figure 4C). The growth phase dependency of the expression of SaeR-dependent genes was also observed on the mRNA level in Figure 2B. Fibrinogen-binding proteins (Ecb and Efb), coagulase (Coa), superantigen-like protein 11 (Ssl11), and immunoglobulin-binding protein (Sbi) are important for innate immune evasion during early stages of infection.^95–98^ During later stages, production of pore-forming toxins such as hemolysins (Hla and HlgBC) and production of secreted enzymes such as serine proteases (e.g., SplB and SplC) are increased.^99,100^

Next, we investigated the effect of IsrR on the SaeR regulon expression. Irrespective of their occurrence in different growth phases (Figure 4B,C), we observed higher levels of the Sae-dependent proteins in the strains expressing *isrR* (ratio of mean OD_540nm_-normalized maxLFQ values IsrR-positive/IsrR-negative: 2.1; paired Wilcoxon test: *p*-value = 2.23 × 10^–14^). However, we noted that some proteins, such as Emp, Ssl9, Ssl7, SelX, and LukD, showed no change in abundance in response to *isrR* expression (Figure 4A). For these proteins, we also observed that the deletion of the Sae system had only minor effects on their levels, at least under the conditions of our study. Thus, IsrR positively affected the protein levels of strictly SaeR-dependent regulon members covered by our analysis (Figure 4B,C), which is consistent with the assumption that IsrR has a positive effect on Sae activity. In contrast, no consistent effect of IsrR on the virulence regulators AgrA/RNAIII^101,102^ and SarA^103^ could be observed (Figure S10).

Of note, the influence of IsrR on Sae-dependent proteins could indeed explain the previously described enhanced virulence factor production under iron-limited conditions.^29^ This was confirmed by our study, where the HG001 wild-type under iron-limited conditions showed higher levels of Sae-dependent proteins than the HG001 pJLctrl strain under iron-sufficient conditions (Figure 4) (ratio of mean OD_540nm_-normalized maxLFQ values HG001 under iron-limited conditions/HG001 pJLctrl under iron-sufficient conditions: 1.2; paired Wilcoxon test: *p*-value = 0.0105).

### IsrR Causes Sae-Dependent and Sae-Independent Changes of the Secretome

To address additional effects of IsrR on the secretome, we used an unbiased ROPECA statistical analysis and compared protein levels of *isrR*-expressing and nonexpressing strains (Figures 3A, S11 and Table S10). For the identification of IsrR-dependent proteins (Figure 5), we followed the workflow introduced in Ganske *et al.* (2024).^19^ Differentially abundant proteins between the *isrR*-expressing and nonexpressing strains were considered as candidates if they showed an absolute fold change of at least 1.5 and *q*-value of less than 0.05 in at least one of the two growth phases. The protein candidate sets derived from the individual comparisons, i.e., the iron-limitation condition, the constitutive *isrR* expression in the HG001 wild-type background, and the constitutive *isrR* expression in the Δ*sae* background, were combined (Figure 5A). Of particular note, we included the Δ*saePQRS* mutant containing the *isrR* expression plasmid compared to the empty vector control in order to address, in particular, Sae-independent effects of IsrR.

**Figure 5:**
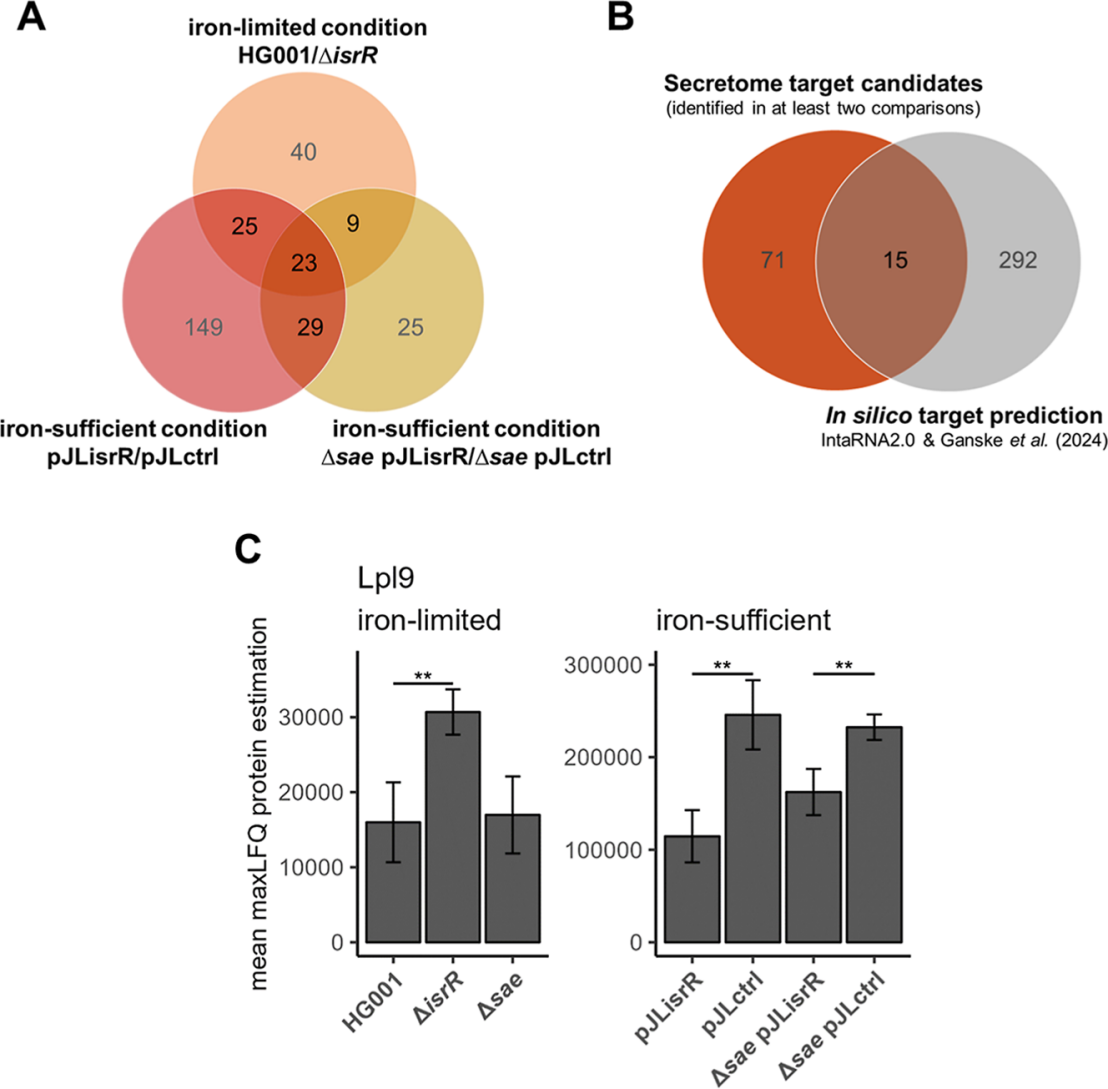
Overview of IsrR-driven effects on the secretome of . (A) Identification of IsrR targets based on the changes of protein abundances in the secretome. For each of the comparisons, candidates were considered if they were significantly altered (|fold change| > 1.5 and *q*-value <0.05) in at least one growth phase and if they were not discordantly altered between the two growth phases. Candidates identified in at least two comparisons were considered as secretome target candidates. (B) Integration of the 86 identified secretome target candidates with in silico IsrR target prediction. Predictions according to IntaRNA2.0 ^56^ specifically for NCTC 8325 (Table S7) and Ganske *et al.* (2024)^19^ were considered. (C) Protein levels of Lpl9 in the stationary growth phase as an example of a protein pattern representing negative IsrR regulation. The bar chart depicts the amount (mean maxLFQ protein level) of Lpl9 between IsrR-expressing (HG001, pJLisrR, and Δ*sae* pJLisrR) and nonexpressing (Δ*isrR*, pJLctrl, and Δ*sae* pJLctrl) strains. Error bars represent the standard deviation of the biological replicates. Statistics: Welch-*t* test on protein levels (*p* < 0.001: ***, *p* < 0.01: **, and *p* < 0.05: *).

This approach resulted in 86 proteins with significantly altered protein levels in at least two of the three comparisons (Table S11). For 15 of the 86 IsrR-affected proteins, an mRNA–IsrR interaction was predicted (Figure 5B). Based on the protein patterns, 13 of the 15 potential targets are affected negatively, and two of them are affected positively by IsrR (Figure 5C and Table S11). Of the 13 negatively affected proteins, five proteins (Ndh2b, AcsA, SdhA, SdhB, and NarG) correspond to known IsrR targets (Table 1). The remaining known IsrR targets (Table 1) are missing in the secretome analysis because cytoplasmic and membrane proteins are only partially covered. In addition to the five known targets, the secretome approach newly revealed ten potential IsrR targets (Table 3). Of these, Lpl9 (Figure 5C) and SAOUHSC_02074 are secreted proteins with lower abundance in the presence of IsrR, which likely correspond to targets regulated through the classical mode of sRNA action, i.e., inhibition of translation by obstructing the ribosome-binding site.^104^ The *lpl9* gene is located in the so-called lipoprotein-like cluster at the νSaα genomic island. The *lpl* cluster is immune stimulatory and contributes to invasion of into host cells.^105^

**Table 3:** Newly Identified Potential IsrR Targets

locus tag	gene symbol^37^	protein localization^38^	description
Negative Targets			
SAOUHSC_00405	*lpl9*	extracellular	uncharacterized lipoprotein
SAOUHSC_01008	*purE*	cytoplasmic	5-(carboxyamino)imidazole ribonucleotide mutase
SAOUHSC_01812	*pde2*	cytoplasmic	DHH/DHHA1 domain-containing phosphodiesterase
SAOUHSC_01910	*pckA*	cytoplasmic	phosphoenolpyruvate carboxykinase
SAOUHSC_02074		extracellular	phi PVL orf 39-like protein
SAOUHSC_02139	*pncA*	cytoplasmic	pyrazinamidase/nicotinamidase
SAOUHSC_02373		cytoplasmic	l-aspartate–l-methionine ligase
SAOUHSC_03022		cytoplasmic	UPF0312 protein
Positive targets			
SAOUHSC_00436	*gltD*	cytoplasmic	glutamate synthase subunit beta
SAOUHSC_01676	*floA*	cytoplasmic Membrane	flotillin-like protein

The flotillin FloA is potentially positively regulated by IsrR. FloA is a membrane scaffold protein promoting spatial interaction of proteins with functional membrane microdomains.^106^ It contributes to virulence^107^ and assembly of the type VII secretion system-mediated secretion of virulence factors.^108^

Of the set of 86 IsrR-dependent proteins (Figure 5A), 29 proteins were positively and 57 were negatively affected. Of the 29 proteins positively affected by the presence of IsrR, 20 are localized extracellularly or associated with the cell wall, which could indicate a general trend in which IsrR is a positive regulator of secreted proteins. These 20 proteins are indirectly regulated by IsrR and include 12 proteins belonging to the SaeR regulon. Among the other eight secreted proteins positively affected by IsrR were the virulence factors serine-aspartate repeat proteins SdrD and SdrC^109,110^ and staphopain ScpA.^111^

The serine protease-like proteins SplB, SplE, and SplF encoded by the SaeR-regulated *splABCDEF* operon showed higher levels in the presence of IsrR not only in the HG001 wild-type but also in the Δ*sae* mutant when comparing the Δ*sae* pJLisrR strain to the Δ*sae* pJLctrl strain. In agreement, the trend could also be observed for all identified Spl proteins (SplB, SplC, SplD, SplE, and SplF) (Figure 4A). Serine protease-like proteins (e.g., SplB and SplC) are important immunomodulatory factors^112,113^ and are also involved in the distribution of bacteria in the infected tissue.^114^ The observed effect on Spl protein levels could indicate that IsrR also regulates Spl protein levels by an Sae-independent mechanism. Indeed, *splA* is a predicted IsrR target.^19^ For this target, the suggested interaction site with IsrR sRNA is not located at the ribosome binding site. This is a common feature of positive regulation by sRNAs, for example, *via* transcript stabilization.^115^ A similar effect of IsrR was proposed for the *sirTM* operon (SAOUHSC_00304, *gcvH-L*, SAOUHSC_00306, *sirTM*, and *lplA2*). Here, only SAOUHSC_00304 is a predicted IsrR target, but all proteins encoded by the operon possess an IsrR-dependent pattern.^19^ In line with the previous findings, GcvH-L and LplA2 were also identified as positively IsrR-affected proteins in the secretome (Table S11).

### IsrR Influences the Protein Levels of the Heme and Siderophore Uptake Systems

In our approach to identify IsrR-affected proteins, 40 proteins were detected solely under iron-limited conditions (Figure 5A). This is the natural condition in which the Fur-dependent *isrR* gene is expressed, and thus, its regulatory effect is most relevant under this condition.^116,117^ In addition, a fundamental adjustment of the secretome under iron-restricted conditions is known,^90,118^ and IsrR targets specific to this conditions are missing in the experiments based on constitutive expression of *isrR* under iron-sufficient conditions.

 uses several iron uptake systems to acquire iron in the host environment (reviewed in Hammer and Skaar (2011)^119^ and Conroy *et al.* (2019)^120^): the iron-regulated surface determinants form the heme uptake system IsdABECDEF in , and siderophore-based acquisition is achieved by the siderophore uptake systems HtsABC (specific to staphyloferrin A), SirABC (staphyloferrin B), FhuBGC-D1/D2 (hydroxymate-type siderophores), and SstABCD (catechol-type siderophores). However, is only able to synthesize the siderophores staphyloferrin A and B using the Sfna and Sbn pathway, respectively.^121,122^ In addition, synthesis and uptake of the wide-spectrum metallophore staphylopine is mediated by the Cnt pathway.^123^

Interestingly, proteins that are part of the heme uptake system, namely, IsdB, IsdC, IsdD, IsdG, and IsdH,^124,125^ showed significantly lower abundance in the IsrR-deficient strain (Table S10). Moreover, IsdA and IsdE were also positively affected by the presence of IsrR (Figure 6A). In line with this, the effect on the heme uptake system was already observed in the cellular proteome^19^ (Figure S12A).

**Figure 6:**
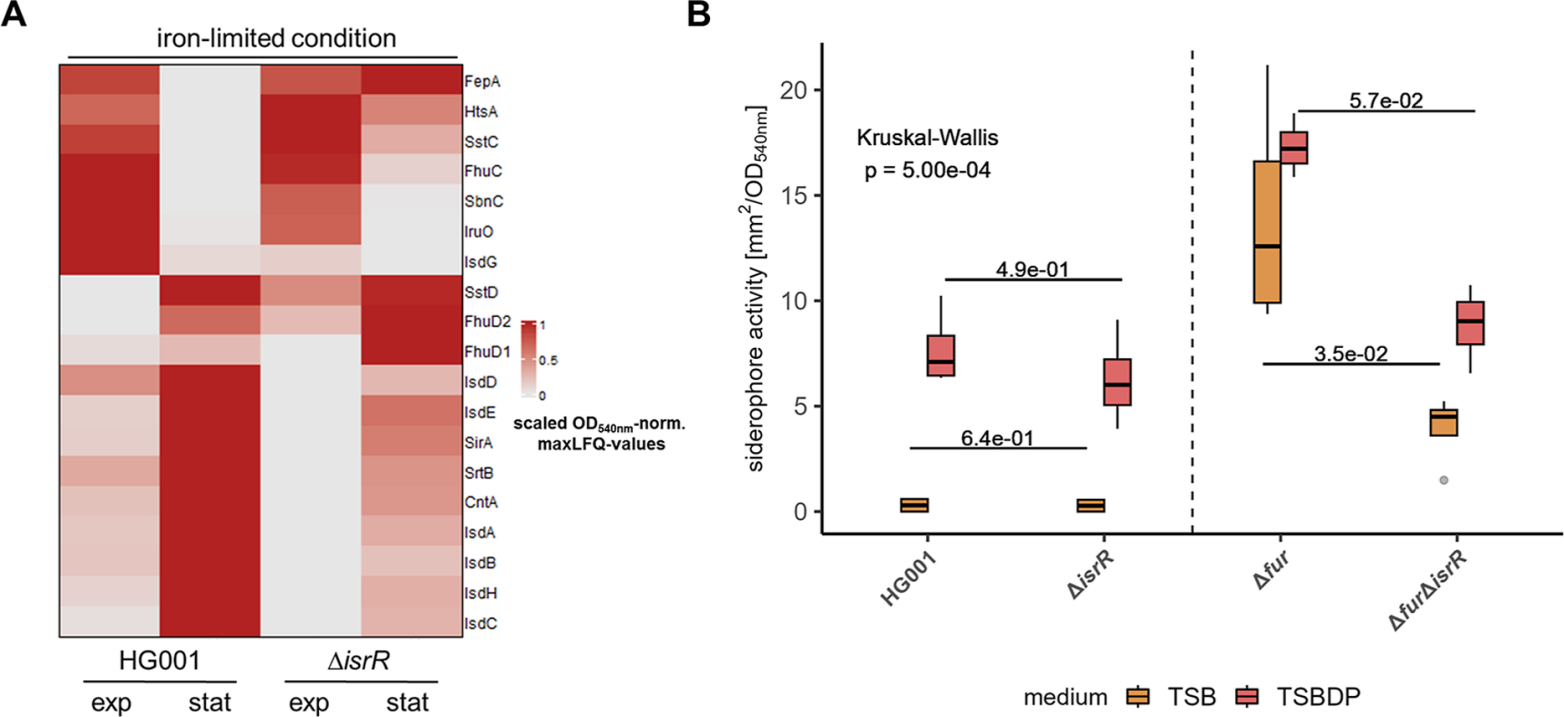
IsrR positively influences the protein levels of the heme uptake system and impacts the siderophore-mediated iron uptake of *.* (A) Heat map of Fur regulon proteins identified in the secretome. MaxLFQ values were normalized to the mean OD_540nm_ per condition at a harvest time point of the supernatant sample and the min–max scaled per protein. (B) CAS-Agar diffusion assay to determine siderophore activity. Strains were cultivated in TSB or TSBDP, and the supernatant was harvested in stationary growth phase. Sterile filtered supernatant was applied to CAS agar plates. Siderophore activity was determined based on the area of orange iron–poor complex formation around the spot. Siderophore activity was subsequently normalized to OD_540nm_. Boxplots represent the median of four biological replicates. Statistics: Kruskal–Wallis test on siderophore activity and Wilcoxon test with Benjamini–Hochberg *p*-value adjustment as post hoc test. Results of relevant post hoc pairwise comparisons are depicted.

In addition to the heme uptake system, also several compounds of the siderophore/metallophore-based iron uptake systems, were altered in abundance. In particular, CntA and SirA were present in lower amounts in the secretome of the Δ*isrR* strain (Figure 6A and Table S10). CntA and SirA are involved in the staphylopine^123^ and staphyloferrin B uptake,^126^ respectively. Strikingly, in the cellular proteome, the corresponding biosynthetic pathways followed the same IsrR-dependent pattern (Figure S12B,C). However, proteins involved in iron acquisition were not predicted to be IsrR targets except for IsdG^19^ (Table S7).

Next, we asked if the observed IsrR-driven effect on metallophore-based iron uptake proteins is also relevant for the efficacy of iron acquisition. Indeed, we could show that the siderophore activity determined *via* the CAS agar diffusion assay was reduced in a Δ*fur*Δ*isrR* strain compared to a Δ*fur* strain under iron-rich and iron-limited conditions and to a lesser extent also in the Δ*isrR* strain compared to the HG001 wild-type under iron-limited conditions (Figure 6B). This is in line with the data reported by Rios-Delgado *et al.* (2025).^23^

## Discussion

The importance of the Fur-regulated sRNA IsrR for bacterial fitness was recently demonstrated, for example, by regulation of the TCA cycle,^19,22,23^ nitrate respiration,^18^ and oxidative stress response.^19^

Coronel-Tellez *et al.* (2022)^18^ and Rios-Delgado *et al.* (2025)^23^ demonstrated the relevance of IsrR for pathogenicity in mice. In the study presented here, we show that IsrR is involved in the regulation of virulence factors, which certainly contributes to the importance of IsrR for infections. We describe the positive effect of IsrR on the expression of *hla*, which is regulated by the SaeRS two-component system. Furthermore, we show that IsrR positively affects Sae activity and thus the protein levels of members of the SaeR regulon, a large group of staphylococcal virulence factors.

The data presented indicate that the IsrR-driven effect on the SaeR regulon is most likely due to the modulation of Sae activity by IsrR. Stimuli of the Sae system range from environmental signals such as subinhibitory levels of antibiotics, pH, and high concentrations of sodium chloride^82,127^ to host-related signals such as hydrogen peroxide as a phagocytosis-related signal and human neutrophil peptides such as α-defensin.^79,128,129^

However, because was not exposed to any of those external stimuli in our study, IsrR likely modulates the basal level of Sae activity. One possibility would be that IsrR targets an Sae modulatory protein. The options are (i) the WalKR-regulated protein LyrA (SpdC), which interacts with the membrane-bound sensor kinase SaeS and leads to activation of the system,^130,131^ (ii) the lipoprotein CamS which inhibits Sae activity,^132^ and (iii) the small protein ScrA which is involved in the activation of the Sae system likely *via* destabilization of the cell membrane.^133,134^ A second possibility for IsrR-dependent modulation of Sae activity lies in the global metabolic changes that were described in previous studies (Table 1) and confirmed here (Table S11). In particular, IsrR causes down-regulation of the TCA cycle (*citB*, *sucA*, *sucB*, *sdhC*, *sdhA*, *sdhB*, *mq*o, and *citZ*), respiratory chain components (*sdhC*, *sdhA*, *sdhB*, *ndh2b*, *mqo*, and *qoxA*), and anaerobic nitrate respiration (*narG*, *nasD*, and *narH*). Interestingly, impaired cellular respiration as well as disruption of molybdenum synthesis, which is essential for nitrate respiration,^135^ is shown to increase SaeRS activity.^136,137^ In this context, it is interesting to note that IsrR not only targets enzymes that catalyze nitrate respiration but could also influence molybdenum availability, as the protein levels of the molybdenum ABC transporter subunit ModA were negatively affected by IsrR (Table S11).

With regard to the link between respiration and Sae-dependent virulence of , a role of fatty acid metabolism was recently discussed.^138^ An altered NAD+/NADH ratio resulting from inactivation of the respiratory NADH dehydrogenase (*ndh2*) genes leads to free fatty acid accumulation, which negatively affects SaeRS activity.^138^ High levels of exogenous and intracellular free fatty acids can inhibit the Sae system, most probably by affecting membrane integrity and in turn SaeS kinase activity.^139–141^ On the other hand, an increase in membrane anteiso branched-chain fatty acid (BCFA) levels by induction of BCFA synthesis leads to higher SaeS activity and induction of the SaeR regulon.^142,143^

Irrespective of the molecular mechanism, this study shows that IsrR is an additional player in the regulation of SaeR activity. By this, low iron levels are perceived by as an indicator for the host environment and serves as a trigger for virulence factor expression. Intriguingly, excess of iron inhibits Sae activity, whereas calprotectin, a protein promoting nutritional immunity of the host by mainly sequestering Zn and Mn ions, increases the activity of the Sae system^5,144^ as shown here for IsrR.

The activation of the Sae system *via* *isrR* expression leads to the production of several virulence factors (Figures 1, 2 and 4), which are involved in adherence to the host tissue and cells, expansion into the host tissue, and immune evasion.^36^ Especially, the strong regulation of Hla and other hemolysins and cytotoxins is likely biologically important under iron-limited conditions. Hemolysis is discussed as a means of nutrient and iron acquisition for the bacterium,^145,146^ and erythrocytes can serve as a sole iron source for and other staphylococcal species.^30,147^ For growth on iron acquired from hemolysis, the hemoglobin-binding proteins IsdBH and the heme uptake system are required,^30^ demonstrating the close connection of the SaeR-regulated hemolysis and the iron limitations response. Interestingly, heme availability inhibits the Sae system,^67^ and a link between Sae activity and *isdAB* expression was also observed.^148,149^

Our data demonstrate that IsrR positively influences the protein abundance of the heme uptake system (Figure 6). Furthermore, our findings and the work of Rios-Delgado *et al.* (2025)^23^ suggest an even broader involvement of IsrR in the regulon of iron acquisition, especially for the staphylopine and staphyloferrin B biosynthesis and uptake systems. The increase of iron uptake systems by an iron-repressed sRNA is also known for, e.g., ,^150,151^ and .^152^

Most likely, IsrR does not directly regulate these iron uptake systems in , as in general no IsrR binding sites were predicted for the respective transcripts. The IsrR impact onto iron uptake systems might be mediated by the IsrR target CcpE.^22^ In addition to its metabolic functions, CcpE regulates the expression of genes encoding the heme uptake system and the staphyloferrin B biosynthesis and uptake system, and it is involved in the repression of siderophore production.^153,154^ Furthermore, IsrR targets *hemA* and *hemY*,^19^ which are part of the heme biosynthesis pathway.^155,156^ Sufficient levels of intracellular heme were suggested to repress the staphyloferrin B production through heme-binding by the moonlighting protein SbnI.^157,158^ In addition, as IsrR is involved in intracellular iron homeostasis and increases the intracellular free iron pool,^23^ these differences in available iron could possibly influence Fur and PerR activity^159–161^ as both regulators respond to intracellular iron levels.^162–165^ Fur-mediated regulation also depends on the recently described iron-binding Fur protein antagonist Fpa (formerly YlaN).^166^ Lastly, the protein abundance of iron uptake systems might be dependent on their interaction partners and proper localization: the heme-specific permease IsdF is located in functional membrane microdomains, and its proper localization and activity depends on colocalization with flotillin A.^167^ The presented study indicated that *floA* is an IsrR target (Figure 5 and Table 3). Additionally, a second newly suggested IsrR target is *lpl9* encoding a lipoprotein. Of note, several lipoproteins are involved in iron uptake, namely, SirA, FhuD1, FhuD2, IsdE, SstD, and FepA,^168^ and correct processing of lipoproteins is critical for the growth of under iron-limited conditions.^169^

In the present study, we demonstrated that IsrR is more than the regulator of the iron sparing response in by reducing the synthesis of nonessential iron-containing proteins (Figure 7). Besides its role in metabolic remodeling upon iron limitation,^18,19,22,23^ it links the iron limitation response to virulence, which is a common theme in bacterial pathogens.^13^

**Figure 7:**
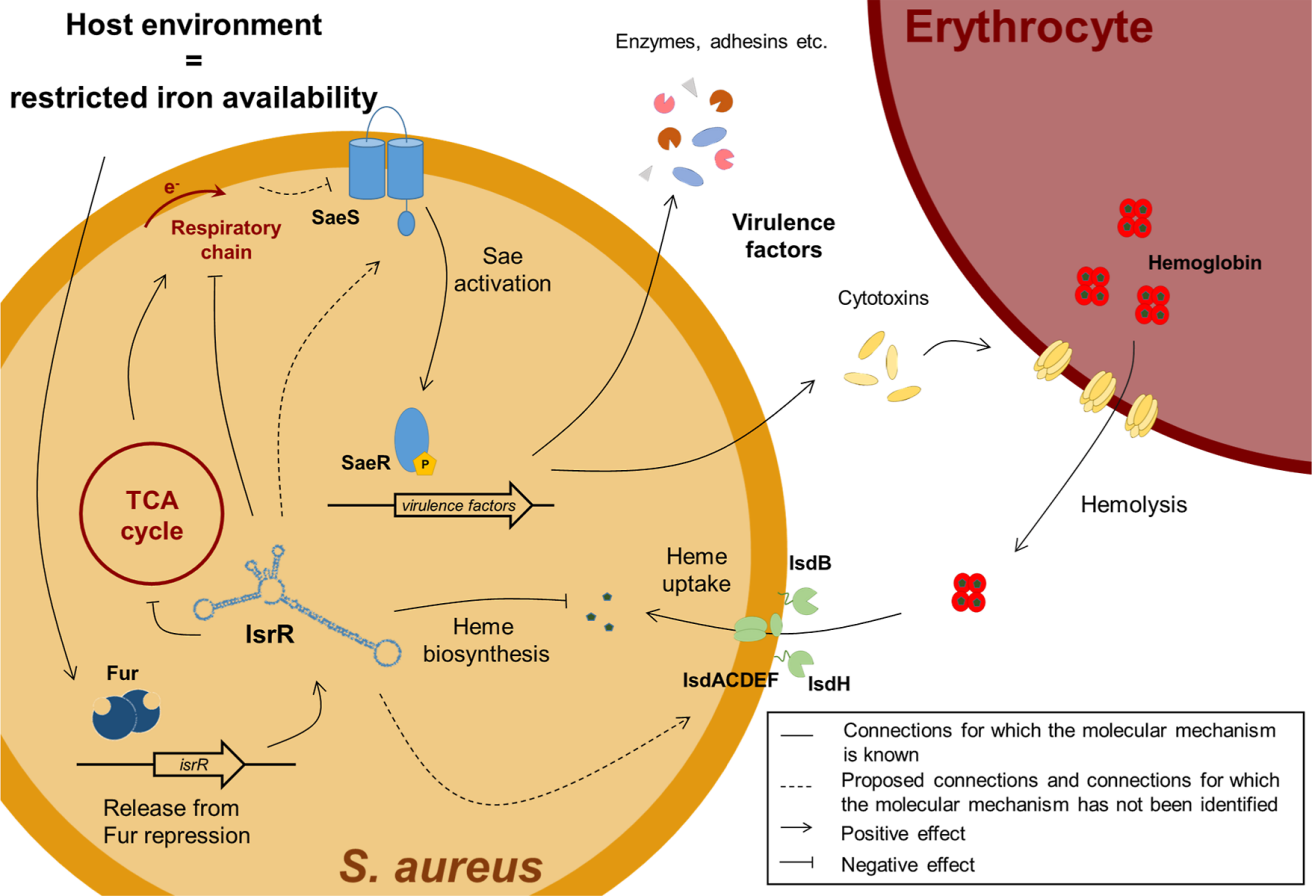
Working model of IsrR linking SaeR-regulated virulence and the iron limitation response in *S. aureus*.

## Supplementary Material













## Data Availability

Secretome data are available *via* ProteomeXchange with identifier PXD055092. Strains will be made accessible upon request.

## Notes

Method S1 - ^15^N external standard; Table S1 - Oligonucleotides used for Northern blot probe generation; Table S2 - Reversed-phase liquid chromatography; Table S3 - Mass spectrometry; Table S4 - Spectronaut^TM^ parameters used for data analysis of mass spectrometry data; Table S5 - R packages; Table S6 - SaeR regulon; Figure S1 - Effect of IsrR on virulence factor transcription under infection-relevant oxygen- and iron-limited conditions; Figure S2 - Effects of *hla*, *saePQRS*, *isrR*, and *fur* deletion on hemolysis activity; Figure S3 - SaeR regulon enrichment analysis in proteome data of Ganske *et al.* (2024); Figure S4 - SaePRS cellular protein levels according to Ganske *et al.* (2024); Figure S5 - Growth curves of the strains cultivated for the secretome analysis; Figure S6 - Enrichment of proteins in the secretome fraction compared to the cellular fraction; Figure S7 - Comparison of detected protein intensities by localization between cellular proteome and secretome samples; Figure S8 - Display of the most abundant proteins detected in the secretome samples; Figure S9 - Ion intensities post normalization; Figure S10 - Heat map of protein abundances of known virulence factors in the secretome and response to the growth phase, the *isrR* expression, and Sae deficiency; Figure S11 - SaeR regulon enrichment analysis in the secretome analysis; Figure S12 - IsdACE cellular protein levels according to Ganske *et al.* (2024) (PDF)
Table S7 - NCTC 8325 genome-wide *in silico* prediction of IsrR targets using IntaRNA2.0 (XLSX)
Table S8 - NCTC 8325 genome-wide *in silico* prediction of protein localizations using DeepLocPro (XLSX)
Table S9 - Complete list of proteins of interest for the secretome analysis (XLSX)
Table S10 - ROPECA-based statistics of differential abundant proteins in the secretome (XLSX)
Table S11 - Complete list of putative IsrR targets (XLSX)
