# Supplementary material for: Linking Virulence and Iron Limitation Response in : The sRNA IsrR Is Involved in SaeRS Activation
Source: J Proteome Res. 2025 Jun 2;24(7):3324–42. doi: 10.1021/acs.jproteome.5c00059 (PMC12235713; doi:10.1021/acs.jproteome.5c00059)
Supplement: Supplementary file 1 [file pr5c00059_si_001.pdf]

Supplemental Data for

# Linking virulence and iron limitation response in *Staphylococcus aureus*: The sRNA IsrR is involved in SaeRS activation

Larissa M. Busch[1], Alexander Ganske[1], Sebastian Reißel[1], Lisa Bleul[2], Christian Hentschker[1], Hannes Wolfgramm[1], Leif Steil[1], Manuela Gesell Salazar[1], Marc Schaffer[1], Alexander Reder[1], Stephan Michalik[1], Christiane Wolz[2], Kristin Surmann[1], Uwe Völker[1], Ulrike Mäder[1]

[1] University Medicine Greifswald, Interfaculty Institute for Genetics and Functional Genomics, Department of Functional Genomics

[2] University of Tübingen, Interfaculty Institute of Microbiology and Infection Medicine

# Supplemental Data Index

Supplemental Data Index ........ 1

1 Supplementary Methods ........ 3

Method S1. $^{15}N$ external standard. ........ 3

2 Supplementary Tables ........ 3

Table S1. Oligonucleotides used for Northern blot probe generation. ........ 3

Table S2. Reversed phase liquid chromatography (RPLC). ........ 4

Table S3. Mass spectrometry. ........ 5

Table S4. Spectronaut$^{TM}$ parameters used for data analysis of mass spectrometry data. 6

Table S5. R packages. ........ 9

Table S6. SaeR regulon. ........ 10

Table S7. ........ 12

Table S8. ........ 12

Table S9. ........ 12

Table S10. ........ 12

Table S11. ........ 12

3 Supplementary Figures ........ 13

Figure S1. ........ 13

Figure S2. ........ 14

Figure S3. ........ 15

Figure S4. ........ 16

Figure S5. ........ 17

Figure S6. ........ 19

Figure S7. ........ 20

Figure S8. ........ 21

Figure S9. .......................................................................................................... 22

Figure S10. .......................................................................................................... 23

Figure S11. .......................................................................................................... 24

Figure S12. .......................................................................................................... 25

4 REFERENCES .......................................................................................................... 26

# 1 Supplementary Methods

## Method S1. $^{15}N$ external standard.

The heavy labelled $^{15}N$ protein standard was generated using *Bacillus subtilis* BSB1 cells [S1]. *B. subtilis* was precultivated in a 1:10 dilution series in BioExpress® Bacterial Cell Media (U-$^{15}N$, 98%; Cambridge Isotope Laboratories Inc., USA) overnight at 37°C. The next morning, a main culture using the same medium was inoculated to an initial $OD_{540nm}$ of 0.05. The entire culture volume was harvested at $OD_{540nm}$ = 1.5 in the exponential growth phase. The cells were pelleted by centrifugation (4 °C, 8,500 x g, 3 min) and the pellets were washed with 20 mM 2-[4-(2-hydroxyethyl)piperazin-1-yl]ethanesulfonic acid (HEPES) pH 8.0. For mechanical disruption of bacterial cells, frozen pellets were suspended in 20 mM HEPES. Then, suspended samples were treated in a bead mill (Retsch GmbH, Germany; 3 min, 30 Hz) and the bacterial cell powder was resuspended in 20 mM HEPES with sodium dodecyl sulfate (SDS) to a final concentration of 1% SDS. The lysates were treated with Pierce™ Universal Nuclease (Pierce, Thermo Fisher Scientific, USA; 2.5 U, 4 mM $MgCl_2$). To remove cell debris, samples were centrifuged (30 min, 17,000 x g, RT). Determination of the protein concentration was also carried out using the Micro BCA™ Protein Assay Kit (Pierce, Thermo Fisher Scientific, USA) and analyzed as in [S2].

# 2 Supplementary Tables

## Table S1. Oligonucleotides used for Northern blot probe generation.

| Probe | Primer name | Sequence (5' → 3')[a] |
|---|---|---|
| *adh1* | SA_ adh1_for | TGAGAGCAGCAGTTGTAACG |
| | SA_ adh1_revT7 | GAAATTAATACGACTCACTATAGGGAGAGCATCAGCACCTAATTCTTTCG |
| *chp* | SA_chp_for | ATGAAAAAGAAATTAGCAACAACAG |
| | SA_chp_revT7 | GAAATTAATACGACTCACTATAGGGAGA TTAGTATGCATATTCATTAG |
| *coa* | SA_coa_for | ATGAAAAAGCAAATAATTTCGCTAG |
| | SA_coa_revT7 | GAAATTAATACGACTCACTATAGGGAGA CCTGTAGTTCATTGTATTC |

| | | |
|---|---|---|
| *hla* | SA_hla_for | CACGTATAGTCAGCTCAGTAAC |
| | SA_hla_revT7 | GAAATTAATACGACTCACTATAGGGAGAGGTCCCCAATTTTGATTCACC |
| *pflB* | SA_pflB_for | GTTAGAAACAAATAAAAATCATGC |
| | SA_pflB_revT7 | GAAATTAATACGACTCACTATAGGGAGACTGACATTTCTGTAGACATC |
| *saeP* | SA_saeP_for | ATGAATACAAAATATTTTTTAGCAG |
| | SA_saeP_revT7 | GAAATTAATACGACTCACTATAGGGAGA TTATTTTAATTTAGCGCCGC |
| *isrR* | SA_isrR_for | TCACTAATGTATAATAGTAGTTG |
| | SA_isrR_revT7 | GAAATTAATACGACTCACTATAGGGAGA AGT GTC GTA AGG GTT TAC TG |

[a] Die Sequenz GAAATTAATACGACTCACTATAGGGAGA$[N]_x$ entspricht der T7-Promotorsequenz

## Table S2. Reversed phase liquid chromatography (RPLC).

| ***Instrument*** | **Ultimate 3000 RSLC (Thermo Scientific)** |
|---|---|
| *Trap column* | 75 µm inner diameter, packed with 3 µm C18 particles (Acclaim PepMap100, Thermo Scientific) |
| *Analytical column* | Accucore 150-C18, (Thermo Fisher Scientific)<br>25 cm x 75 µm, 2,6 µm C18 particles, 150 Å pore size |
| *Buffer system* | binary buffer system consisting of 0.1% acetic acid in HPLC-grade water (solvent A) and 100% ACN in 0.1% acetic acid (solvent B) |
| *Flow rate* | 300 nl/min |
| *Gradient* | 0min-2% B<br>2min-5% B<br>10min-7% B<br>70min-25% B<br>75min-40% B<br>77min-90% B<br>83min-90% B<br>85min-2% B<br>95min-2%B |
| *Column oven temperature* | 40°C |

Table S3. Mass spectrometry.

| | |
|---|---|
| ***Instrument*** | *Orbitrap Exploris 480* |
| ***Electrospray*** | Nanospray Flex™ Ion Source |
| ***Operation mode*** | data-independent |
| ***Full Scan Properties*** | |
| *MS scan resolution* | 120000 |
| *AGC target* | 3e6 (300%) |
| *Maximum ion injection time for the MS scan* | 60 ms |
| *Scan range* | 350 to 1200 m/z |
| *Microscans* | 1 |
| *Polarity* | Positive |
| *RF Lens* | 50% |
| *Spectra data type* | Profile |
| ***DIA Properties (MS2)*** | |
| *Resolution* | 30,000 |
| *Maximum ion injection time for the MS/MS scans* | Auto |
| *Normalized AGC target* | 3E6 |
| *Spectra data type* | Profile |
| *Microscans* | 1 |
| *Isolation window* | 66 |
| *Isolation window width* | 13 m/z |
| *Window overlay* | 2 m/z |
| *Fixed first mass* | 200 |

**Table S4.** Spectronaut$^{TM}$ parameters used for data analysis of mass spectrometry data.

| ***Parameter*** | **Setting** |
|---|---|
| ***Calibration*** | |
| *Calibration Mode* | Automatic |
| *RT Regression Type* | Local (Non-Linear) Regression |
| ***Identification*** | |
| *Pvalue Estimator* | Kernel Density Estimator |
| *Precursor Qvalue Cutoff* | 0.001 |
| *Protein Qvalue Cutoff* | 0.01 |
| *Decoy method* | Mutated |
| *Decoy Limit Strategy* | Dynamic |
| ***Library Filters*** | |
| *Fragment Ions* | |
| *Ion AA Length* | N=3 |
| *m/z* | Min: 200 Max: 3000 |
| *Precursors* | |
| *Best N Fragments per Peptide* | Min: 3 Max: 6 |
| ***Protein Inference*** | |
| *Inference Algorithm* | IDPicker |
| ***Quantification*** | |
| *Precursor Filtering* | Identified (Qvalue) |
| *Imputation Strategy* | Use Background Signal |
| *Quantity MS Level* | MS2 |
| *Quantity Type* | Area |
| *Cross-Run Normalization* | True |
| *Normalization Filter Type* | Library Name Filter |

| | |
|---|---|
| *Library Name* | 20230811_Bacillus_heavy_lib_fix_mod |
| *Normalization Strategy* | Global Normalization (Median) |
| *Row Selection* | Identified in all Runs (Complete) |
| *Interference Correction* | True |
| *Only Identified Peptides* | True |
| *Exclude All Multi-Channel Interferences* | True |
| *MS1 Min* | 2 |
| *MS2 Min* | 3 |
| ***Workflow*** | |
| *Multi-Channel Workflow Definition* | From Library Annotation |
| *Profiling Strategy* | iRT Profiling |
| *Profiling Target Selection* | Profile only non-identified Precursors |
| *Unifiy Peptide Peaks Strategy* | Select corresponding Peak |
| ***XIC Extraction*** | |
| *XIC Extraction Window* | Dynamic |
| *MS1 & MS2 Mass Tolerance Strategy* | Dynamic |
| ***Library set up*** | |
| ***230831_AG_LB_SR_Library_HG001_SN_Iron*** | |
| *Software version* | 18.2.230802.50606 |
| *Digest Rule* | Trypsin/P |
| *Digest Type* | Specific |
| *Missed Cleavage* | 2 |
| *Min Peptide Length* | 7 |
| *Max Peptide Length* | 52 |
| *Toggle N-terminal M* | True |
| *Protein & Peptide FDR* | 0.01 |

| | |
|---|---|
| *Fragment Ions per Peptide* | 6-10 |
| *Protein Database* | Saureus_NCTC8325_aug2023_aureowiki_Lysostaphin_Benzonase_Trypsin_ErmB_ErmC_RepC_Cm_RsbU.fasta |
| ***Library set up*** ***20230811_Bacillus_heavy_lib_fix_mod*** | |
| *Software version* | 18.1.230626.50606 |
| *Digest Rule* | Trypsin/P |
| *Digest Type* | Specific |
| *Missed Cleavage* | 2 |
| *Min Peptide Length* | 7 |
| *Max Peptide Length* | 52 |
| *Toggle N-terminal M* | True |
| *Protein & Peptide FDR* | 0.01 |
| *Fragment Ions* | 6-10 |
| *Protein Database* | 2021_01__uniprot_sp_Bsubtilis_168_incl_isoforms.fasta |

Table S5. R packages.

| Package | Version | Reference |
|---|---|---|
| Tidyverse | 2.0.0 | [S3] |
| FactoMineR | 2.4 | [S4] |
| Ggpubr | 0.6.0 | Alboukadel Kassambara (2023). ggpubr: 'ggplot2' Based Publication Ready Plots |
| Ggrepel | 0.9.5 | Kamil Slowikowski (2021). ggrepel: Automatically Position Non-Overlapping Text Labels with 'ggplot2'. |
| Ggtext | 0.1.2 | Claus O. Wilke and Brenton M. Wiernik (2022). ggtext: Improved Text Rendering Support for 'ggplot2'. |
| Iq | 1.9.6 | [S5] |
| Openxlsx | 4.2.5 | Philipp Schauberger and Alexander Walker (2023). openxlsx: Read, Write and Edit xlsx Files. |
| Patchwork | 1.2.0 | Thomas Lin Pedersen (2024). patchwork: The Composer of Plots. |
| PECA | 1.30.0 | Tomi Suomi, Jukka Hiissa and Laura L. Elo (2021). PECA: Probe-level Expression Change Averaging. |
| Readr | 2.1.4 | Hadley Wickham, Jim Hester and Jennifer Bryan (2023). readr: Read Rectangular Text Data. |
| Readxl | 1.4.3 | Hadley Wickham and Jennifer Bryan (2023). readxl: Read Excel Files. |
| Rstatix | 0.7.2 | Alboukadel Kassambara (2023). rstatix: Pipe-Friendly Framework for Basic Statistical Tests. |
| ComplexHeatmap | 2.15.4 | [S6] |
| RColorBrewer | 1.1-3 | Erich Neuwirth (2022). RColorBrewer: ColorBrewer Palettes. |
| Fgsea | 1.20.0 | [S7] |
| Gghalves | 0.1.4 | Frederik Tiedemann (2022). gghalves: Compose Half-Half Plots Using Your Favourite Geoms. |
| Vroom | 1.6.5 | Jim Hester, Hadley Wickham and Jennifer Bryan (2023). vroom: Read and Write Rectangular Text Data Quickly. |

Table S6. SaeR regulon.

| Locus tag | pan gene symbol | Description |
|---|---|---|
| SAOUHSC_00182 | SAOUHSC_00182 | hypothetical protein |
| SAOUHSC_00192 | *coa* | Staphylocoagulase |
| SAOUHSC_00354 | *selX* | enterotoxin-like toxin X |
| SAOUHSC_00392 | *ssl7* | superantigen-like protein 7 |
| SAOUHSC_00394 | *ssl9* | superantigen-like protein 9 |
| SAOUHSC_00399 | *ssl11* | superantigen-like protein 9 |
| SAOUHSC_00400 | SAOUHSC_00400 | FKLRK protein |
| SAOUHSC_00402 | *lpl3* | uncharacterized lipoprotein |
| SAOUHSC_00717 | *saeP* | DM13 domain-containing protein |
| SAOUHSC_00716 | *saeQ* | DoxX family protein |
| SAOUHSC_00715 | *saeR* | response regulator |
| SAOUHSC_00714 | *saeS* | sensor histidine kinase |
| SAOUHSC_01110 | *ecb* | fibrinogen-binding protein-like protein |
| SAOUHSC_01114 | *efb* | fibrinogen-binding protein |
| SAOUHSC_01115 | *scc* | staphylococcal complement inhibitor |
| SAOUHSC_01121 | *hla* | alpha-hemolysin |
| SAOUHSC_01942 | *splA* | serine protease SplA |
| SAOUHSC_01941 | *splB* | serine protease SplB |
| SAOUHSC_01939 | *splC* | serine protease SplC |
| SAOUHSC_01938 | *splD* | serine protease SplD |
| SAOUHSC_01936 | *splE* | serine protease SplE |
| SAOUHSC_01935 | *splF* | serine protease SplF |
| SAOUHSC_01944 | SAOUHSC_01944 | DUF4888 domain-containing protein |
| SAOUHSC_01955 | *lukE* | leukotoxin LukE |
| SAOUHSC_01954 | *lukD* | leukotoxin LukD |
| SAOUHSC_02161 | *eap/map* | MHC class II analog protein |
| SAOUHSC_02160 | *map_2* | MAP domain-containing protein |
| SAOUHSC_02167 | *scn* | staphylococcal complement inhibitor |
| SAOUHSC_02171 | *sak* | Staphylokinase |
| SAOUHSC_02243 | *lukH* | uncharacterized leukocidin-like protein 2 |
| SAOUHSC_02241 | *lukG* | uncharacterized leukocidin-like protein 1 |

| | | |
|---|---|---|
| SAOUHSC_02706 | *sbi* | immunoglobulin G-binding protein Sbi |
| SAOUHSC_02709 | *hlgC* | leukocidin s subunit |
| SAOUHSC_02710 | *hlgB* | leukocidin f subunit |
| SAOUHSC_02169 | *chp* | chemotaxis-inhibiting protein CHIPS |
| SAOUHSC_02803 | *fnbA* | fibronectin-binding protein A |
| SAOUHSC_02802 | *fnbB* | fibronectin binding protein B |
| SAOUHSC_00816 | *emp* | extracellular matrix and plasma binding protein |
| SAOUHSC_00818 | *nuc* | Thermonuclease |
| SAOUHSC_02163 | *hlb* | truncated Phospholipase C |
| SAOUHSC_02708 | *hlgA* | gamma-hemolysin h-gamma-II subunit |

**Table S7.** NCTC 8325 genome-wide *in silico* prediction of IsrR targets using IntaRNA2.0 (.xlsx)

**Table S8.** NCTC 8325 genome-wide *in silico* prediction of protein localizations using DeepLocPro (.xlsx)

**Table S9.** Complete list of proteins of interest for the secretome analysis (.xlsx)

**Table S10.** ROPECA-based statistics of differential abundant proteins in the secretome (.xlsx)

**Table S11.** Complete list of putative IsrR targets (.xlsx)

# 3 Supplementary Figures

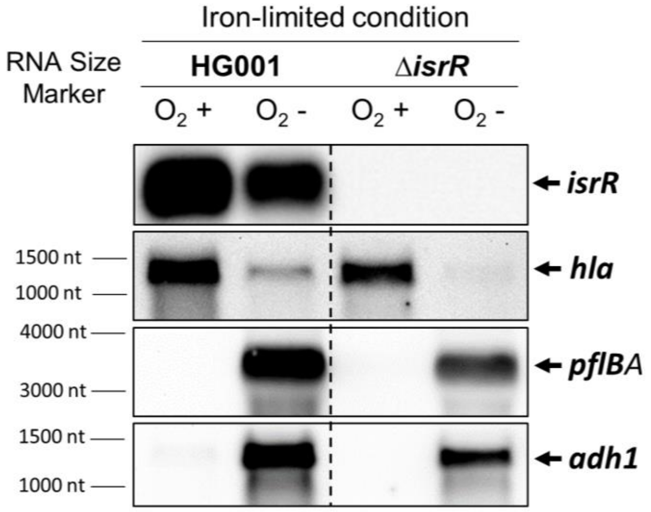


**Figure S1. The effect of IsrR on virulence factor transcription under infection-relevant oxygen- and iron-limited conditions.** Strains were grown under aerobic and anaerobic iron-depleted conditions and samples were harvested in the stationary growth phase (8h after inoculation of the main culture). *S. aureus* HG001 and the isogenic *isrR* mutant (Δ*isrR*) were cultivated. The virulence factor mRNA levels of *hla* were examined. The mRNA levels of the Rex-dependent genes *pflB* and *adh1* serve as markers for anaerobic conditions. IsrR levels serve as control for iron limitation. For each sample, 4 µg of total RNA was loaded per lane. Blots are representatives of at least two independent replicates.

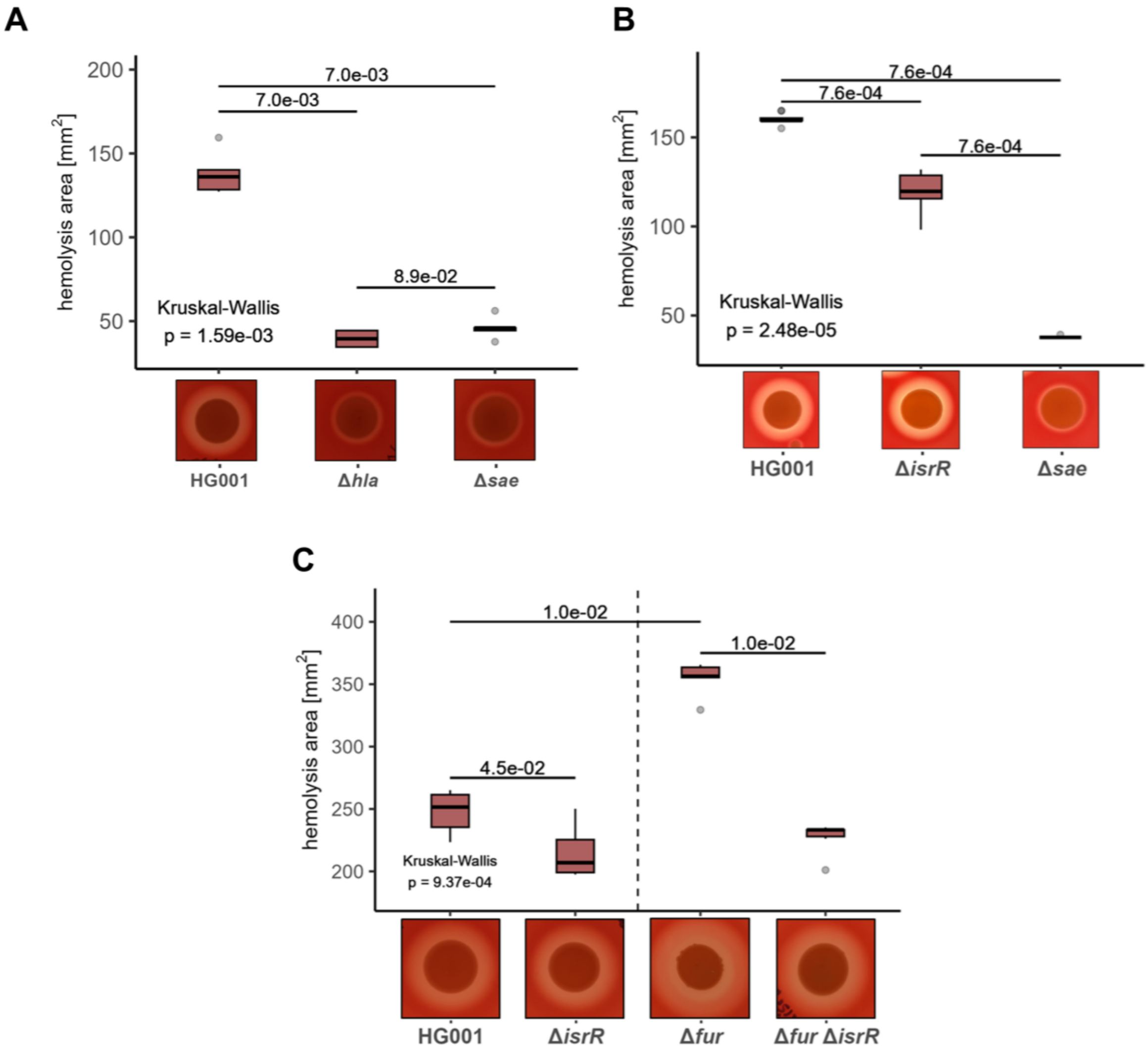


**Figure S2. Effects of *hla*, *saePQRS*, *isrR* and *fur* deletion on hemolysis activity.** Strains were cultivated in TSB at 37°C and at $OD_{540}$ of 1, 10 µl of culture were spotted on 5% sheep blood Colombia agar. Plates were incubated for 24 h (A & B) or 48 h (C). Hemolysis activity was determined based on the area of hemolysis around the spot of growing *S. aureus* cells. Boxplots represent the median of three biological and each two technical replicates. Statistics: Kruskal-Wallis-test on hemolysis activity and Wilcoxon-test with Benjamini-Hochberg p-value adjustment as post-hoc test. Relevant post-hoc pair-wise comparisons are depicted.

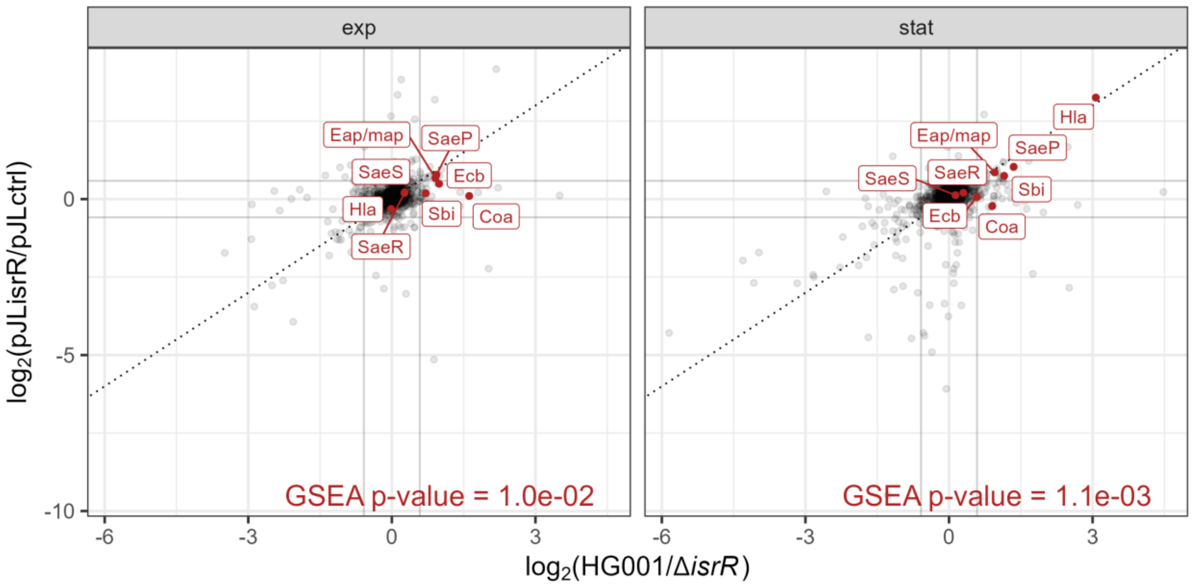


**Figure S3. SaeR regulon enrichment analysis in proteome data of [S8].** Correlation plot of IsrR-driven differences in protein abundances ($log_2$ scale) plasmid carrying strains cultivated under iron-rich conditions and non-plasmid carrying strains cultivated under iron-limited conditions in exponential and stationary growth phase. 1.5-Fold differences are indicated by lines. The dotted line represents a perfect correlation between both experiments. The eight SaeR regulon members identified in both cellular proteome approaches are labeled. Proteins were ranked according to $log_2$ of geometric means of the signal log ratios of both experiments. Gene set enrichment was calculated for the SaeR regulon.

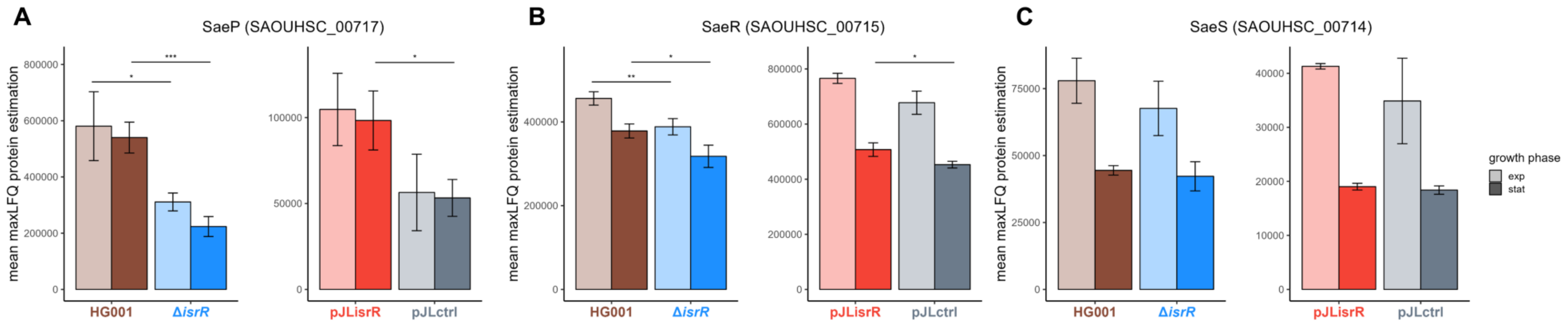


**Figure S4. SaePRS cellular protein levels according to [S8].** The bar charts depict the amount (mean maxLFQ protein level) of indicated proteins in exponential (exp) and stationary (stat) growth phase between *isrR*-expressing (HG001, pJLisrR) and non-expressing strains (Δ*isrR*, pJLctrl). Error bars represent the standard deviation of the four biological replicates. Statistics: Welch-t-test on protein levels ($p < 0.001$: ***, $p < 0.01$: **, $p < 0.05$: *). **(A)** SaeP. **(B)** SaeR. **(C)** SaeS.

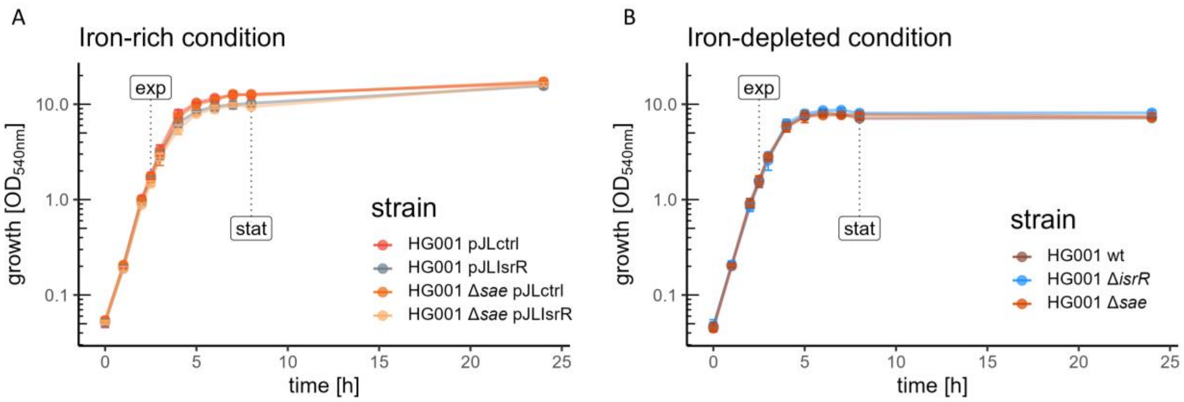


**Figure S5. Growth curves of the strains cultivated for the secretome analysis. (A)** The strains HG001 pJLctrl, HG001 pJLisrR, HG001 Δ*sae* pJLctrl and HG001 Δ*sae* pJLisrR were cultivated in iron-rich TSB and **(B)** the strains HG001, HG001 Δ*isrR* and HG001 Δ*sae* were cultivated in TSBDP. Harvesting time points (2.5h and 8h) are indicated. Each strain was cultivated in four independent biological replicates.

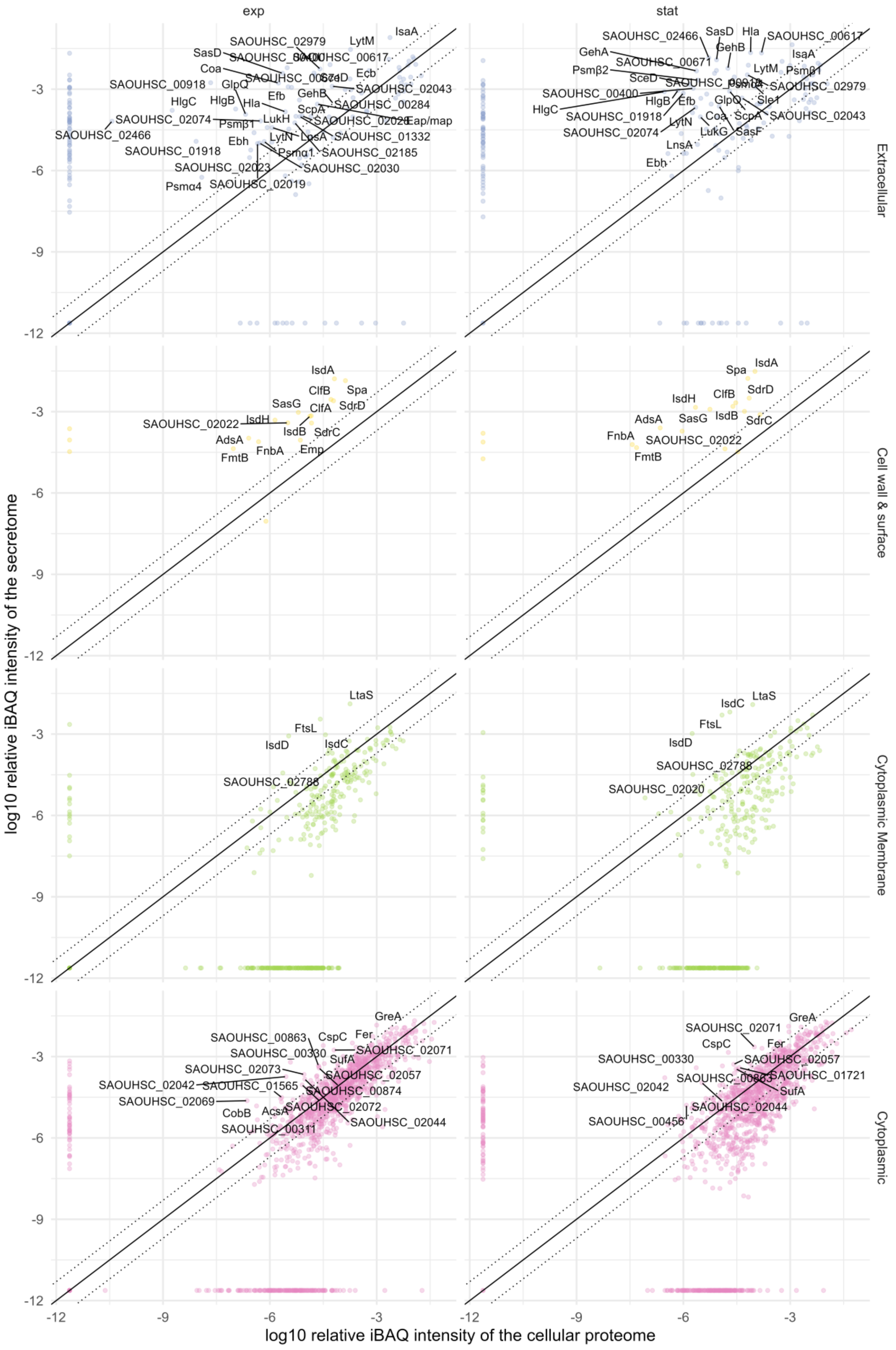
exp
stat
Extracellular
Cell wall & surface
Cytoplasmic Membrane
Cytoplasmic
log10 relative iBAQ intensity of the secretome
log10 relative iBAQ intensity of the cellular proteome
-12
-9
-6
-3

**Figure S6. Enrichment of proteins in the secretome fraction compared to the cellular fraction.** For cellular proteome samples data from [S8] was re-analyzed and compared to the newly-acquired secretome data of the strains HG001, Δ*isrR* and corresponding derivates carrying the pJLctrl and pJLIsrR plasmids. Relative iBAQ values were determined for each sample and subsequently the mean relative iBAQ intensity for each protein was calculated for exponential and stationary growth phase samples. For localization categories, the prediction according to DeepLocPro [S9]. Five-fold difference in relative iBAQ intensities are indicated by the dotted lines. Proteins with ten-fold enrichment in the secretome data were labeled.

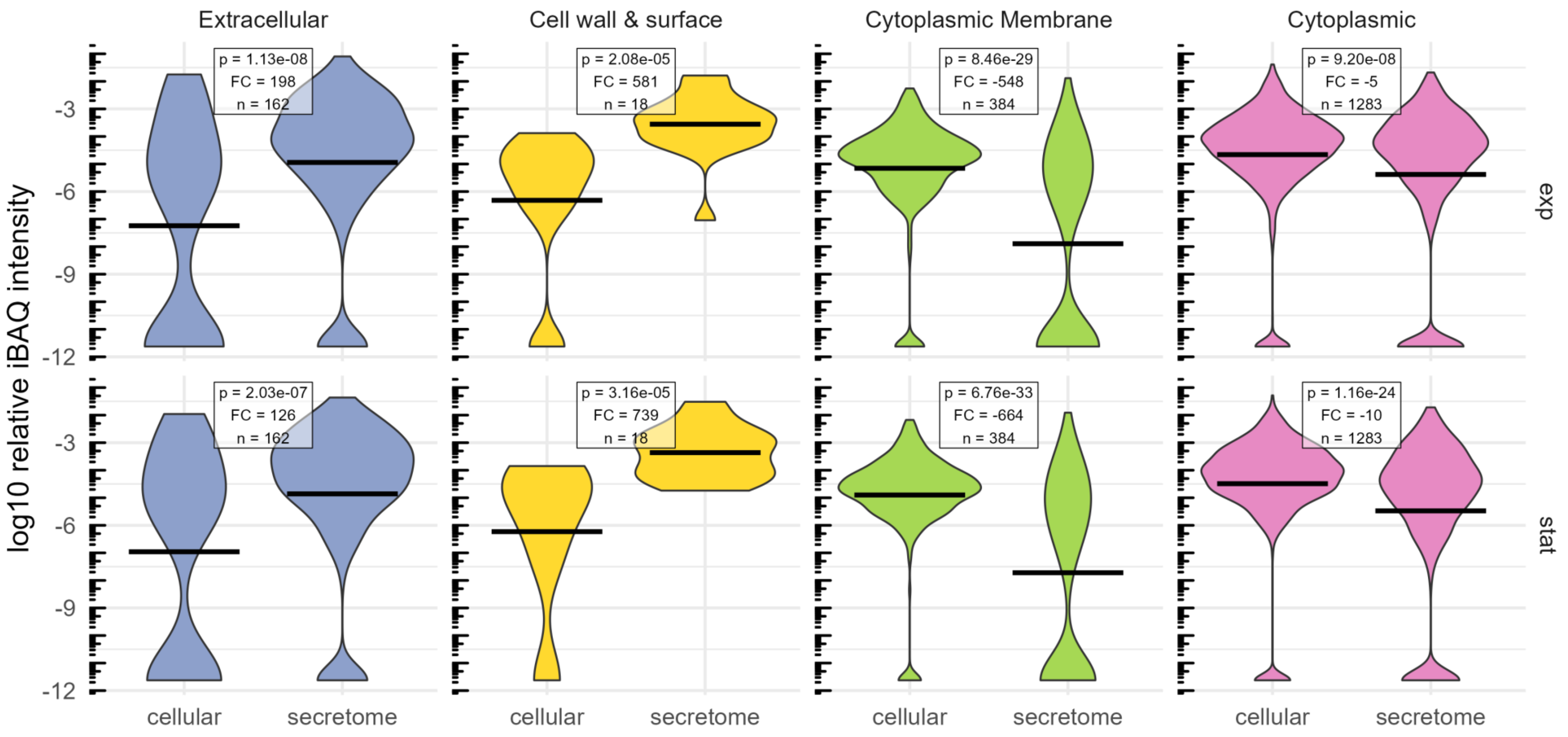


**Figure S7. Comparison of detected protein intensities by localization between cellular proteome and secretome samples.** For cellular proteome samples data from [S8] was re-analyzed and compared to the presented secretome data of the strains HG001, Δ*isrR* and corresponding derivates carrying the pJLctrl and pJLIsrR plasmids. Relative iBAQ values were determined for each sample and subsequently the mean relative iBAQ intensity for each protein was calculated for exponential and stationary growth phase samples. For localization categories, the prediction according

to DeepLocPro [S9]. Differences of intensities respective the localization was testes using the Wilcoxon Rank Sum test (p). The fold change (FC) was calculated based on the mean relative iBAQ intensity ratio of the secretome fraction compared to the cellular fraction.

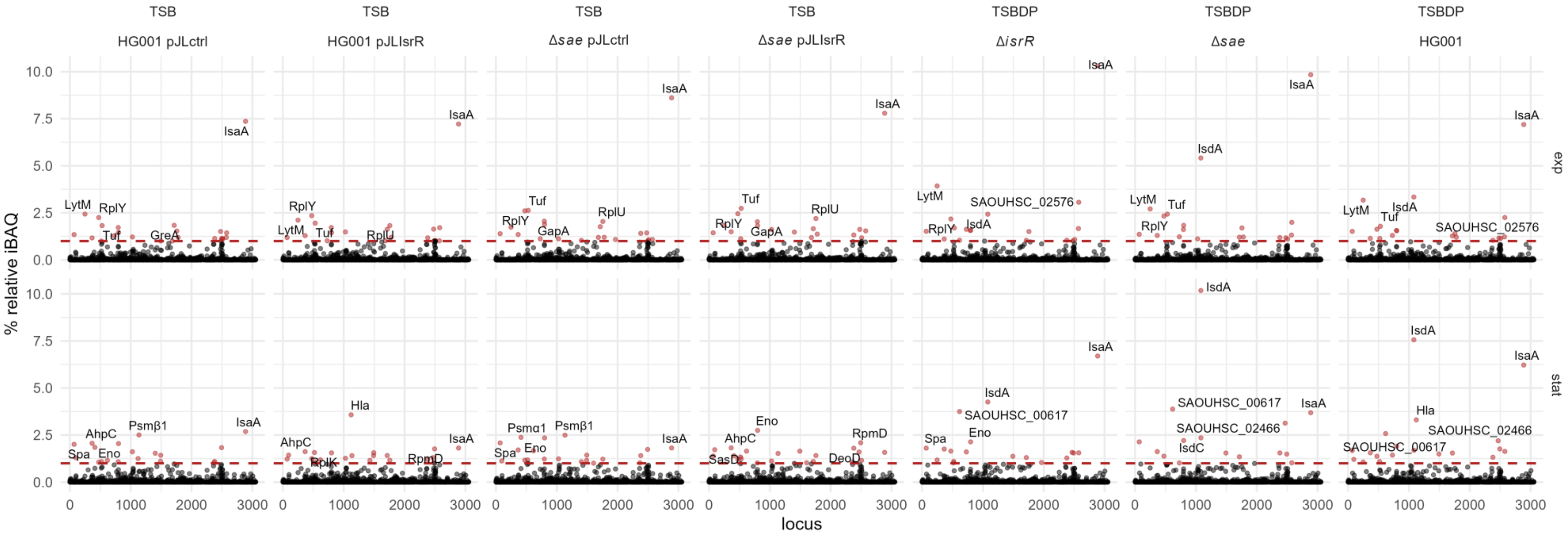


**Figure S8. Display of the most abundant proteins detected in the secretome samples.** The mean relative iBAQ intensities for each protein was determined and plotted against its respective genetic locus. Proteins with a higher relative abundance than 1% are depicted in red. The 1% threshold is indicated as red line. The top five proteins for each condition are labeled.

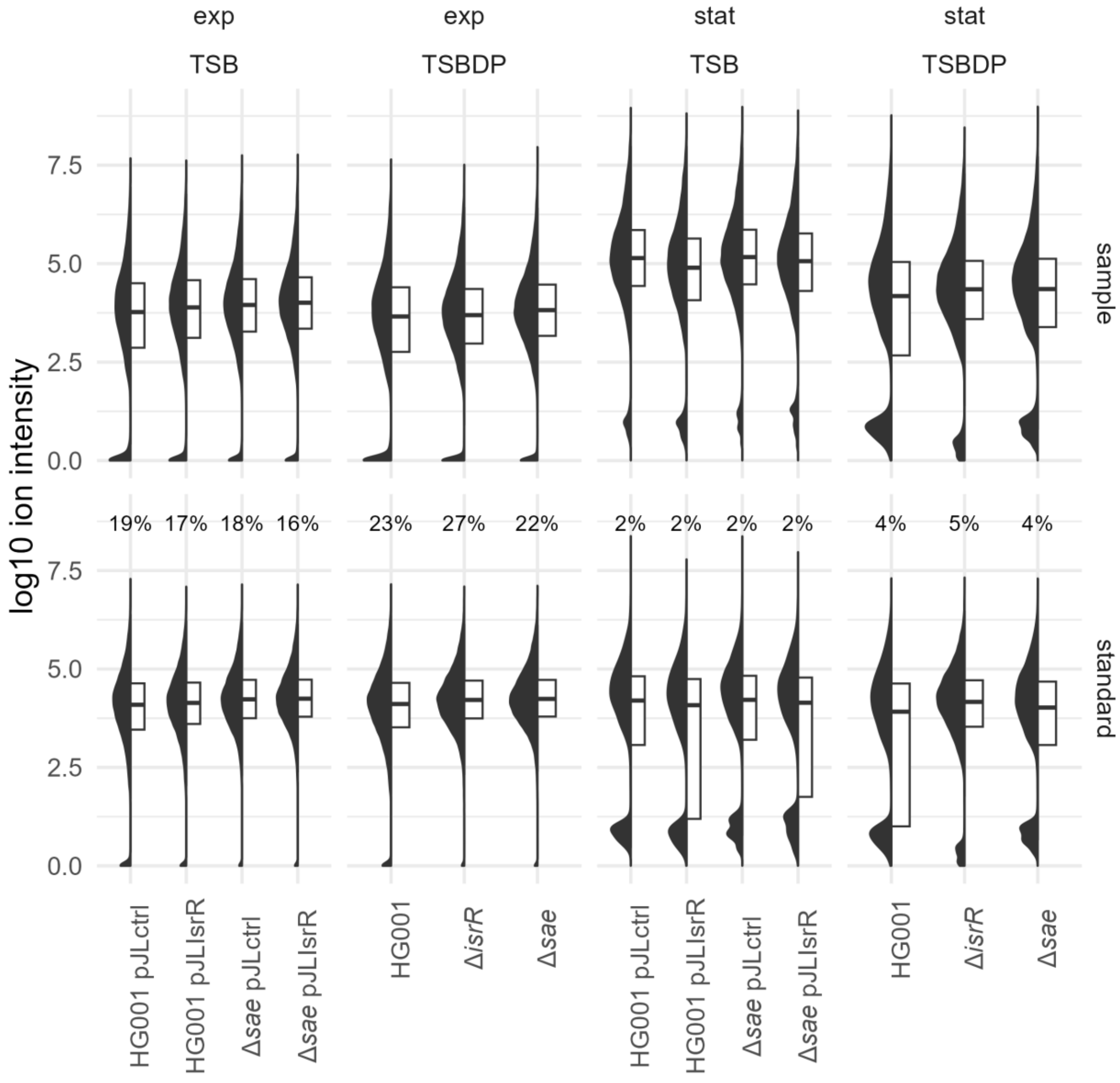


**Figure S9. Ion intensities post normalization.** Ion intensities were global median median-normalized based on the 100%-percentile of the $^{15}N$-standard. Using this approach sample sets of very different overall protein intensity enables robust relative quantification of actual differences in protein abundance. The percentage of the total ion intensity possessed by $^{15}N$-standard ions per condition is indicated as percentage.

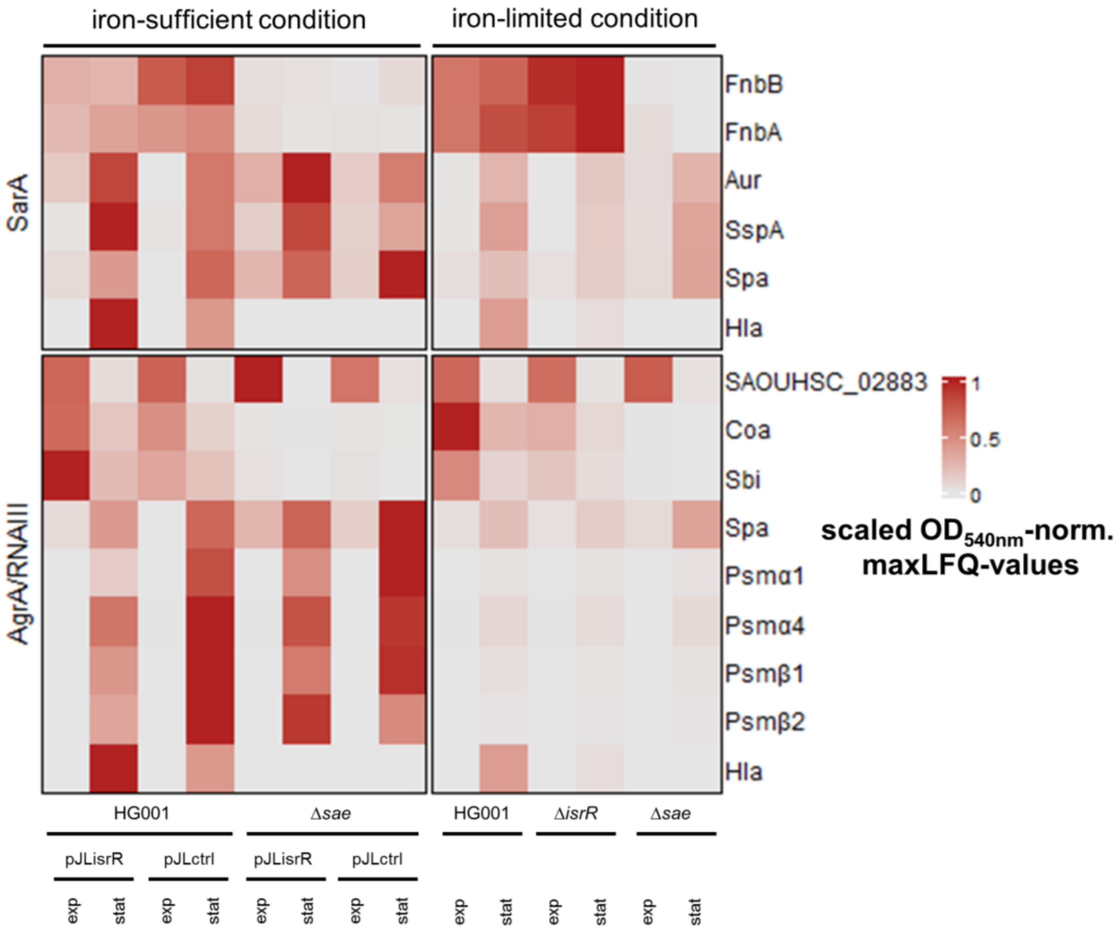


**Figure S10. Heat map of protein abundances of known virulence factors in the secretome and response to the growth phase, the *isrR* expression and Sae deficiency.** MaxLFQ values [S10] were normalized to the mean $OD_{540nm}$ per condition at harvest point of the supernatant sample and min-max scaled per protein. The SarA and AgrA/RNAIII regulon were depicted according to manually curated *Aureo*Wiki regulon lists.

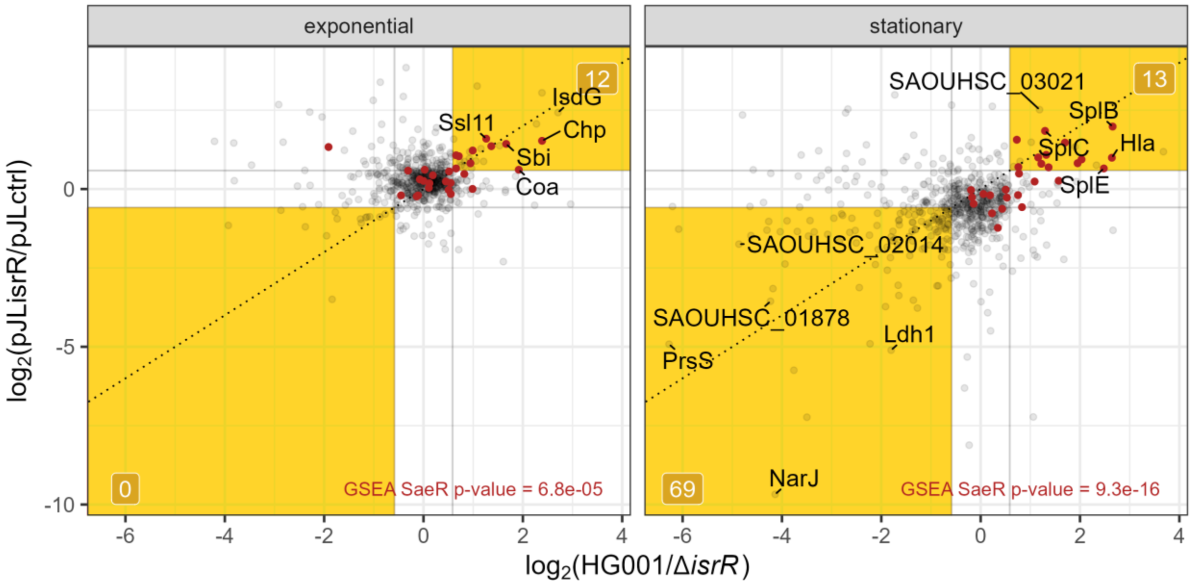


**Figure S11. SaeR regulon enrichment analysis in the secretome analysis.** Correlation plot of IsrR-driven differences in protein abundances ($log_2$ scale) plasmid carrying strains cultivated under iron-rich conditions and non-plasmid carrying strains cultivated under iron-limited conditions in exponential and stationary growth phase. 1.5-Fold differences are indicated by grey lines. The dotted line represents a perfect correlation between both experiments. Yellow boxes indicate consistent significant changes (|fold change|>1.5 in the same direction). Numbers depict the numbers of consistent significant changes with a combined q-value (geometric mean of FDR-adjusted p-values between both conditions). The 28 SaeR regulon members identified in both approaches are labeled. Proteins were ranked according to $log_2$ of geometric means of the signal log ratios of both experiments. Gene set enrichment was calculated for the SaeR regulon.

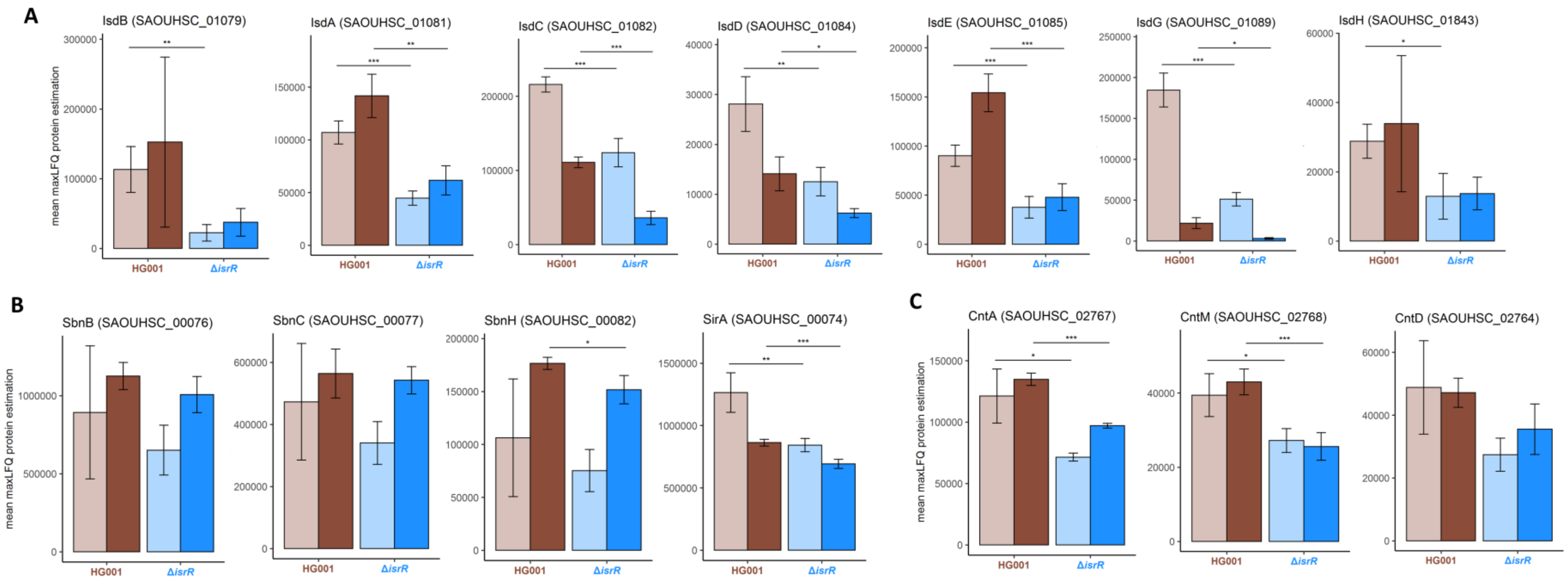


**Figure S12. IsdACE cellular protein levels according to [S8].** The bar charts depict the amount (mean maxLFQ protein level) of indicated proteins in exponential (pale bars) and stationary (solid bars) growth phase between the *isrR*-expressing (HG001) and non-expressing strains (Δ*isrR*). Error bars represent the standard deviation of the four biological replicates. Statistics: Welch-t-test on protein levels ($p < 0.001$: ***, $p < 0.01$: **, $p < 0.05$: *). **(A)** Heme uptake system. **(B)** Staphyloferrin B synthesis and uptake system. **(C)** Staphylopine synthesis and uptake system.

# 4 References

(S1) Nicolas, P.; Mäder, U.; Dervyn, E.; Rochat, T.; Leduc, A.; Pigeonneau, N.; Bidnenko, E.; Marchadier, E.; Hoebeke, M.; Aymerich, S.; Becher, D.; Bisicchia, P.; Botella, E.; Delumeau, O.; Doherty, G.; Denham, E. L.; Fogg, M. J.; Fromion, V.; Goelzer, A.; Hansen, A.; Härtig, E.; Harwood, C. R.; Homuth, G.; Jarmer, H.; Jules, M.; Klipp, E.; Le Chat, L.; Lecointe, F.; Lewis, P.; Liebermeister, W.; March, A.; Mars, R. A. T.; Nannapaneni, P.; Noone, D.; Pohl, S.; Rinn, B.; Rügheimer, F.; Sappa, P. K.; Samson, F.; Schaffer, M.; Schwikowski, B.; Steil, L.; Stülke, J.; Wiegert, T.; Devine, K. M.; Wilkinson, A. J.; van Dijl, J. M.; Hecker, M.; Völker, U.; Bessières, P.; Noirot, P. Condition-dependent transcriptome reveals high-level regulatory architecture in *Bacillus subtilis*. *Science (New York, N.Y.)* **2012**, *335*, 1103–1106.

(S2) Reder, A.; Hentschker, C.; Steil, L.; Gesell Salazar, M.; Hammer, E.; Dhople, V. M.; Sura, T.; Lissner, U.; Wolfgramm, H.; Dittmar, D.; Harms, M.; Surmann, K.; Völker, U.; Michalik, S. MassSpecPreppy-An end-to-end solution for automated protein concentration determination and flexible sample digestion for proteomics applications. *Proteomics* **2024**, *24*, e2300294.

(S3) Wickham, H.; Averick, M.; Bryan, J.; Chang, W.; McGowan, L.; François, R.; Grolemund, G.; Hayes, A.; Henry, L.; Hester, J.; Kuhn, M.; Pedersen, T.; Miller, E.; Bache, S.; Müller, K.; Ooms, J.; Robinson, D.; Seidel, D.; Spinu, V.; Takahashi, K.; Vaughan, D.; Wilke, C.; Woo, K.; Yutani, H. Welcome to the Tidyverse. *Journal of Open Source Software* **2019**, *4*, 1686.

(S4) Lê, S.; Josse, J.; Husson, F. FactoMineR : An R Package for Multivariate Analysis. *Journal of Statistical Software* **2008**, *25*.

(S5) Pham, T. V.; Henneman, A. A.; Jimenez, C. R. iq: an R package to estimate relative protein abundances from ion quantification in DIA-MS-based proteomics. *Bioinformatics (Oxford, England)* **2020**, *36*, 2611–2613.

(S6) Gu, Z. Complex heatmap visualization. *iMeta* **2022**, *1*, e43.

(S7) Korotkevich, G.; Sukhov, V.; Budin, N.; Shpak, B.; Artyomov, M. N.; Sergushichev, A. *Fast gene set enrichment analysis* 3l, 2016.

(S8) Ganske, A.; Busch, L. M.; Hentschker, C.; Reder, A.; Michalik, S.; Surmann, K.; Völker, U.; Mäder, U. Exploring the targetome of IsrR, an iron-regulated sRNA controlling the synthesis of iron-containing proteins in *Staphylococcus aureus*. *Frontiers in microbiology* **2024**, *15*, 1439352.

(S9) Moreno, J.; Nielsen, H.; Winther, O.; Teufel, F. Predicting the subcellular location of prokaryotic proteins with DeepLocPro. *Bioinformatics (Oxford, England)* **2024**, *40*, btae677.

(S10) Cox, J.; Hein, M. Y.; Luber, C. A.; Paron, I.; Nagaraj, N.; Mann, M. Accurate proteome-wide label-free quantification by delayed normalization and maximal peptide ratio extraction, termed MaxLFQ. *Molecular & cellular proteomics* **2014**, *13*, 2513–2526.
